# Alcohol‐Related Liver Disease and Metabolic Dysfunction‐Associated Steatotic Liver Disease: Molecular Pathogenesis and Therapeutic Interventions

**DOI:** 10.1002/mco2.70532

**Published:** 2025-12-07

**Authors:** Yupin Tan, Yirui Hu, Ye Yang, Huikuan Chu

**Affiliations:** ^1^ Division of Gastroenterology Union Hospital Tongji Medical College Huazhong University of Science and Technology Hubei China

**Keywords:** alcoholic liver disease, immune regulation, intestinal microbiota, metabolism‐related fatty liver disease, molecular mechanisms, treatment strategies

## Abstract

Alcohol‐related liver disease (ALD) and metabolic dysfunction‐associated steatotic liver disease (MASLD) are among the most prevalent chronic liver conditions globally, placing a substantial burden on global healthcare systems. Although significant progress has been made in their study, the pathogenic mechanisms remain incompletely defined, and effective treatments are still limited. This review aims to provide a comprehensive analysis of the shared and divergent molecular pathogenic mechanisms underlying these two diseases and to systematically summarize the latest therapeutic intervention strategies. Although ALD and MASLD have distinct etiologies, they share multiple pathophysiological pathways, such as dysregulated lipid metabolism, programmed cell death, cellular senescence, gut dysbiosis, and immune activation. We focus on key molecular events within these shared pathways, such as impaired fatty acid oxidation, increased lipogenesis, activation of pyroptotic and necroptotic signaling pathways, engagement of the p53–p21 senescence axis, and gut microbiota‐driven immune signaling pathways via microbial metabolites and microbe‐associated molecular patterns. Building upon these mechanistic insights, the review further outlines therapeutic strategies targeting lipid metabolism, cell death, cellular senescence, microbiota modulation, and immunomodulation, while also discussing the specific challenges and opportunities. Ultimately, this review proposes a mechanistic framework to guide the development of precision therapies for ALD and MASLD.

## Graphical abstract

Alcohol‐related (ALD) and metabolic dysfunction‐associated (MASLD) steatotic liver disease, while initiated by distinct etiologies (ethanol vs. metabolic stress), converge on shared core pathogenic pathways that drive progression. These include dysregulated lipid metabolism, programmed cell death, cellular senescence, gut dysbiosis, and immune activation. This common mechanistic underpinning explains the overlapping disease phenotypes and opens avenues for unified therapeutic strategies. Consequently, treatment paradigms are evolving beyond initial triggers to target these fundamental processes, with promising approaches including PPAR agonists for metabolism, senolytics for senescence, caspase inhibitors for cell death, microbiota‐directed therapies, and novel immunomodulators. This framework emphasizes that precision management of ALD and MASLD will be guided by targeting convergent pathological mechanisms rather than solely their divergent origins.

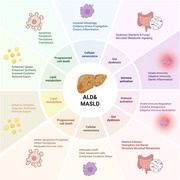

## 1 Introduction

Alcohol‐related liver disease (ALD) and metabolic dysfunction‐associated steatotic liver disease (MASLD) (formerly known as nonalcoholic fatty liver disease [NAFLD]) constitute two major contributors to the rising global burden of liver‐related morbidity and mortality [1]. ALD encompasses a spectrum of histopathological changes, ranging from hepatic steatosis and fibrosis to cirrhosis, potentially culminating in liver failure. Chronic and excessive alcohol consumption remains a leading cause of cirrhosis worldwide and a substantial contributor to liver‐related mortality [2]. Approximately one quarter of cirrhosis deaths are attributable to alcohol. Over the past two decades, alcohol consumption has steadily increased in many countries—particularly in low‐ and middle‐income countries—a trend expected to persist until 2030 [3]. Consequently, the global burden of ALD and its complications, including hepatocellular carcinoma (HCC), is expected to increase [4]. MASLD is closely associated with insulin resistance and metabolic dysfunction [5]. Defined in 2020 to more accurately reflect its metabolic origins, MASLD typically progresses from isolated hepatic steatosis to metabolic dysfunction‐associated steatohepatitis (MASH), eventually advancing to cirrhosis and HCC [6]. The global prevalence of MASLD rose from 25.26% (21.59–29.33) in 1990–2006 to 38.00% (33.71–42.49) in 2016–2019—an increase of 50.4% (*p* < 0.001)—with projections indicating continued growth [7]. In the United States, MASH has emerged as a leading indication for liver transplantation in adults, particularly among those undergoing transplantation for HCC. The burden of MASLD and its complications—including HCC—is expected to rise further in the coming years [8]. Taken together, ALD and MASLD constitute a major global health challenge, imposing substantial economic and healthcare burdens through their contributions to progressive fibrosis, cirrhosis, and liver cancer [1].

Historically, ALD and MASLD were regarded as discrete disease entities with well‐characterized etiologies. However, accumulating evidence has identified substantial histological and mechanistic parallels between the two conditions. This convergence has important clinical implications, particularly given the frequent coexistence of harmful alcohol consumption in individuals with obesity and type 2 diabetes. This overlap has prompted the conceptualization of MetALD (steatotic liver disease with both MASLD and increased alcohol intake risk factors), further underscoring the critical importance of investigating their shared pathogenic pathways [9].

Contemporary research into the pathogenesis of ALD now emphasizes the molecular‐level understanding. Accumulating evidence indicates that ethanol and its metabolites, particularly acetaldehyde, disrupt hepatic lipid metabolism, induce oxidative stress (OxS), and impair mitochondrial function in hepatocytes [10]. Concurrently, alcohol compromises the intestinal barrier, leading to gut dysbiosis and translocation of microbial products (e.g., lipopolysaccharide [LPS]). These events subsequently activate hepatic innate immune responses and downstream proinflammatory signaling [11]. Moreover, cellular senescence and programmed cell death modalities (e.g., apoptosis, pyroptosis) are increasingly recognized as central mediators of alcohol‐induced liver injury.

In MASLD, systemic insulin resistance is the principal metabolic driver, promoting increased de novo lipogenesis (DNL) and diminished fatty acid oxidation, which lead to excessive lipid accumulation within hepatocytes [12]. Analogous to ALD, gut dysbiosis plays a significant role in the “second hit” of MASLD, exacerbating hepatic inflammation and fibrosis via the gut–liver axis [13]. Furthermore, lipotoxicity, OxS, endoplasmic reticulum (ER) stress, and various forms of cell death collectively contribute to the pathological evolution from isolated steatosis to MASH.

Despite distinct etiologies, ALD and MASLD exhibit significant convergence across five fundamental pathogenic pathways: dysregulated lipid metabolism, cell death, cellular senescence, gut dysbiosis, and immune dysregulation. This shared pathophysiology provides a crucial opportunity to understand the common foundations of these diseases and to inform the development of shared therapeutic strategies. Accordingly, this review aims to: (1) systematically elucidate and compare the molecular pathogenesis of ALD and MASLD within these domains; (2) explore the bidirectional interplay between gut microbiota/derived metabolites, cellular senescence, and the immune system in driving liver disease progression; and (3) synthesize recent advances in therapeutic interventions, with particular emphasis on novel approaches targeting these shared molecular pathways.

## 2 Disordered Lipid Metabolism

**FIGURE 1:**
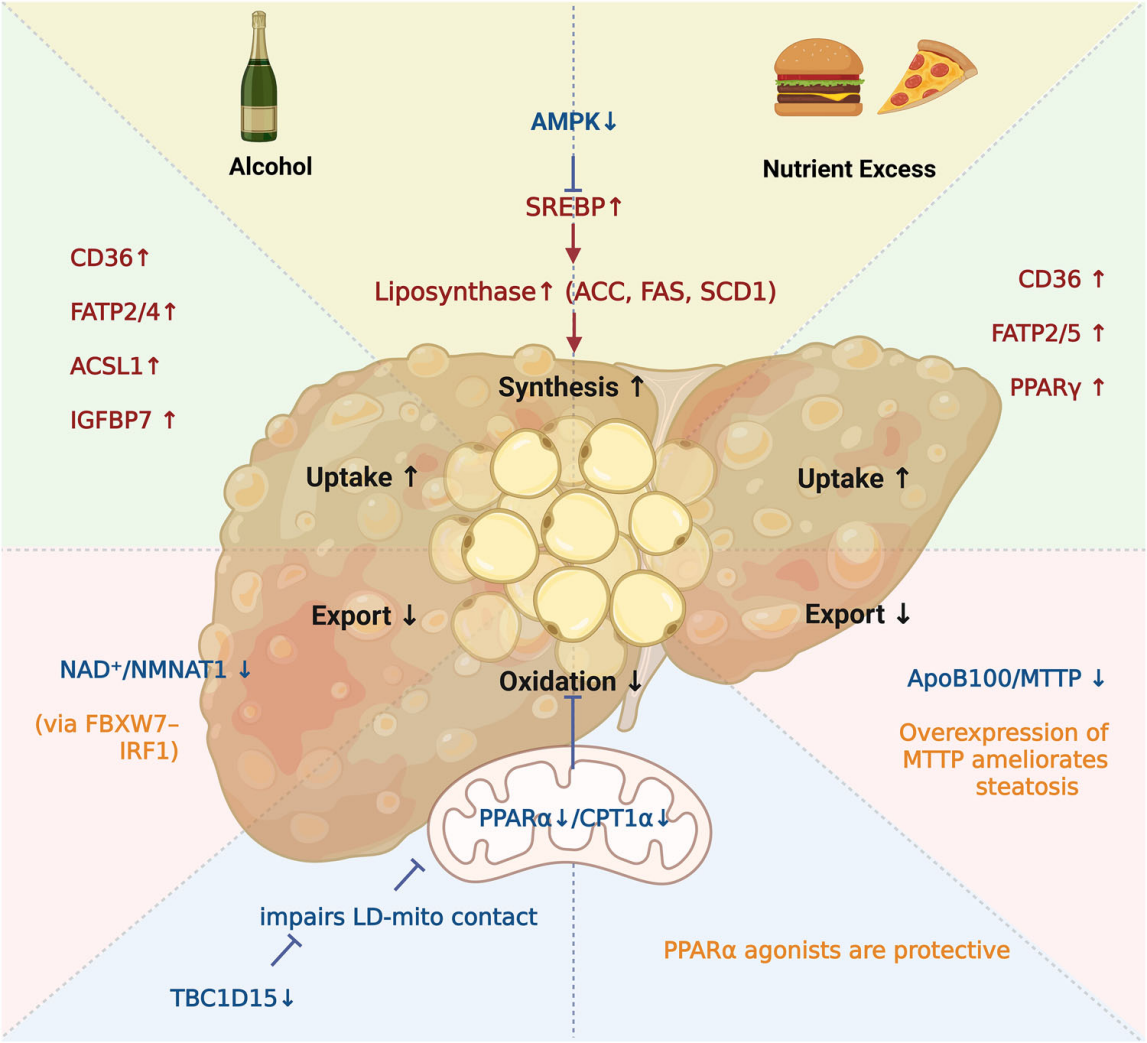
Schematic illustration of disrupted hepatic lipid metabolism in ALD and MASLD. Both ALD and MASLD share a common pathophysiological core of hepatic lipid accumulation (steatosis) driven by an imbalance between four fundamental pathways: (1) increased peripheral fatty acid uptake, (2) enhanced de novo lipogenesis (DNL), (3) impaired fatty acid β‐oxidation, and (4) reduced lipid export. While the initial triggers differ (ethanol metabolism in ALD vs. nutritional excess and insulin resistance in MASLD), the convergence on these pathways leads to excessive triglyceride (TG) accumulation within hepatocytes. In ALD, the imbalance is primarily driven by alcohol‐induced upregulation of fatty acid transporters (CD36, FATPs, ACSL1) and the lipogenic factor SREBP‐1c, coupled with suppression of the oxidative regulator PPARα and the lipid export machinery (e.g., NAD⁺/NMNAT1). In MASLD, the disorder is characterized by PPARγ‐mediated enhancement of fatty acid uptake (via CD36, FATPs), SREBP‐1c/ChREBP‐driven de novo lipogenesis, and defective lipid export due to impaired ApoB100/MTTP function. *Abbreviations*: ACC, acetyl‐CoA carboxylase; ACSL1, acyl‐CoA synthetase long‐chain family member 1; ALD, alcoholic liver disease; ApoB100, apolipoprotein B100; CD36, cluster of differentiation 36; DNL, de novo lipogenesis; FAS, fatty acid synthase; FATP, fatty acid transport protein; IGFBP7, insulin‐like growth factor‐binding protein 7; MASLD, metabolic dysfunction‐associated steatotic liver disease; MTTP, microsomal triglyceride transfer protein; NAD⁺, nicotinamide adenine dinucleotide; NMNAT1, nicotinamide mononucleotide adenylyltransferase 1; PPAR, peroxisome proliferator‐activated receptor; SCD1, stearoyl‐CoA desaturase‐1; SREBP‐1c, sterol regulatory element‐binding protein 1c; TG, triglyceride.

Dysregulated lipid metabolism is a common core pathophysiological feature of both ALD and MASLD, though the underlying triggers and specific mechanisms differ. In ALD, ethanol and its metabolites (e.g., acetaldehyde) disrupt the balance among lipid synthesis, oxidation, and excretion, leading to excessive accumulation of triglyceride (TG) within hepatocytes. Similarly, MASLD is mainly driven by nutritional excess and insulin resistance. However, both disrupt hepatic lipid metabolism through four fundamental pathways: increased peripheral fatty acid uptake, enhanced endogenous lipid synthesis, and impaired fatty acid oxidation and lipid export. These alterations culminate in intrahepatic lipid accumulation, thereby contributing to ALD and MASLD progression (Figure 1).

### 2.1 Increased Peripheral Fatty Acid Uptake

The liver uptakes circulating free fatty acids through fatty acid transport proteins (FATPs). In ALD, hepatic uptake of peripheral fatty acids is significantly increased via upregulation of transporters such as CD36 and FATPs [14]. CD36, a key fatty acid transporter localized to the plasma membrane, is significantly upregulated in ALD and directly mediates the uptake of long‐chain fatty acids [15]. In addition, ER‐localized FATP2 and FATP4, along with mitochondria‐localized ACSL1, functionally contribute to fatty acid uptake by catalyzing fatty acid esterification, among which FATP2 exhibits the most pronounced effect on promoting fatty acid uptake [15]. Moreover, alcohol‐induced overexpression of insulin‐like growth factor binding protein 7 (IGFBP7) further exacerbates hepatic steatosis by suppressing peroxisome proliferative activated receptor, alpha (PPARα) signaling and indirectly modulating the transcription of genes involved in lipid uptake [16]. Meanwhile, studies have demonstrated that in MASH mice, the expression of FATPs is elevated, and their mediated lipid uptake accelerates hepatic steatosis [17]. Knockdown of FATP2 in mice reduces fatty acid uptake, while knockdown of FATP5 reprograms hepatic lipid composition and attenuates MASH progression [17, 18]. Additionally, in MASLD, aberrant upregulation of hepatic PPARγ increases the expression of fatty acid transporters, including fatty acid binding protein 1 (FABP1) and CD36, leading to lipid accumulation in hepatocytes [19, 20, 21]. Similarly, the use of PPARγ antagonists or genetic knockdown of FABP1/CD36 has been shown to ameliorate MASLD [22, 23, 24, 25]. Collectively, these findings strongly suggest that targeting the PPARγ–FABP1/CD36 axis represents a promising strategy to directly reduce pathological lipid uptake at its source.

### 2.2 Enhanced Endogenous Lipid Synthesis

In addition to peripheral fatty acid uptake, the control of hepatic DNL by key lipogenic enzymes such as acetyl‐CoA carboxylase (ACC), fatty acid synthase (FAS), and stearoyl‐CoA desaturase‐1 (SCD1), as well as transcription factors including sterol regulatory element‐binding protein‐1 (SREBP‐1) and carbohydrate response element binding protein (ChREBP), is pivotal for lipid metabolism. In ALD, ethanol metabolism activates SREBP‐1c, leading to upregulation of major lipogenic enzymes including ACC, FAS, and SCD1[26]. This process is epigenetically regulated through alcohol‐mediated downregulation of the histone methyltransferase Setdb1, which alleviates H3K9me3‐based repression of Plin2—a lipid droplet stabilizer—and inhibits its chaperone‐mediated autophagy, thereby promoting Plin2 accumulation and lipid deposition [27]. Arrestin domain‐containing 3, upregulated in ALD, directly binds to and stabilizes SCD1 by attenuating its ubiquitination, thereby promoting lipogenesis [28]. Concurrently, apoptosis‐stimulating protein p53 2 activates DNL through activation of PPARγ signaling, while RNF2, in concert with its interacting partner ubiquitin‐specific protease 7 (USP7), augments lipogenic pathways via the PI3K/AKT pathway [29, 30]. Similarly, studies have shown that the expression of SREBP1 and SCD1 is upregulated in MASLD mice, and knockdown of SCD1 in the livers of high‐fat diet (HFD)‐fed mice reduces hepatic TG accumulation and attenuates steatosis [31, 32]. Furthermore, ChREBP expression is also upregulated in MASLD mice, and its overexpression leads to increased expression of lipogenic genes such as ACC1, FAS, and SCD1, which is positively correlated with the severity of hepatic steatosis [33, 34]. Conversely, ChREBP‐deficient knockout mice exhibit reduced expression of lipogenic genes and decreased hepatic lipid synthesis [35]. These findings collectively indicate that targeting these enzymes and transcription factors to suppress hepatic DNL constitutes a promising therapeutic strategy for MASLD.

### 2.3 Impaired Fatty Acid Oxidation

PPARα is a central regulator of hepatic fatty acid β‐oxidation, and it has been well established that its dysfunction is directly associated with the development and progression of ALD and MASLD [36, 37]. In ALD, ethanol and its metabolites markedly suppress the transcriptional activity of PPARα and its target genes (e.g., carnitine palmitoyltransferase 1A (CPT1A)), resulting in impaired fatty acid β‐oxidation [38]. AMP‐activated protein kinase (AMPK), an energy sensor, exerts bifunctional regulatory roles by inhibiting DNL while promoting fatty acid β‐oxidation, making it a potential therapeutic target [39, 40, 41, 42]. Genetic ablation of IGFBP7 mitigates alcohol‐induced suppression of PPARα and restores lipid homeostasis [16]. Ethanol also downregulates TBC1D15, disrupting mitochondrial–lipid droplet interactions and impairing PKA‐driven nuclear translocation of PLIN5, which subsequently reduces PGC‐1α and CPT1α expression and attenuates mitochondrial β‐oxidation [38]. Quercetin counteracts ethanol‐induced mitochondrial ROS and promotes PGC‐1α‐mediated mitochondrial biogenesis, thereby mitigating OxS and restoring fatty acid oxidation [43]. The impaired capacity for fatty acid β‐oxidation exacerbates the progression of ALD. In patients with MASLD, particularly in the MASH stage, hepatic PPARα levels are significantly reduced and its activity is suppressed, leading to diminished fatty acid oxidation capacity and uncontrolled inflammation, thereby accelerating the progression from steatosis to hepatitis and fibrosis [44]. The use of PPARα agonists has been shown to attenuate hepatic steatosis, ballooning, and inflammatory markers in MASLD mouse models [45]. CPT1α, another rate‐limiting enzyme in fatty acid β‐oxidation regulated by PPARα, has also been demonstrated to reduce hepatic steatosis in mice when its activity is enhanced [46]. These findings collectively indicate that PPARα confers a protective role in MASLD.

### 2.4 Lipid Export

In addition to fatty acid oxidation pathways, the regulation of lipid export is essential for maintaining hepatic lipid homeostasis. Chronic alcohol exposure leads to intracellular TG accumulation by impairing the function of lipid export machinery. For instance, alcohol consumption reduces nuclear nicotinamide adenine dinucleotide (NAD⁺) levels and diminishes NMNAT1 expression and activity, modulated via the FBXW7–IFN regulatory factor (IRF)1 axis, resulting in mitochondrial dysfunction, reduced lipid oxidation, and potentially reduced hepatic lipid secretion—collectively contributing to intracellular lipid accumulation [47]. These regulatory alterations collectively diminish hepatic lipid export capacity, thereby contributing to the progression of ALD. For MASLD, there are two key mediators for liver TG output, namely, apolipoprotein B100 (apoB100) and microsomal TG transfer protein (MTTP). Studies have shown that chronic hepatic lipid overload suppresses apoB100 secretion by inducing ER stress. In patients with MASH, reduced synthesis of apoB100 diminishes lipid transport, thereby exacerbating steatosis [48, 49]. Similarly, in MASH mouse models, overexpression of MTTP has been shown to lower hepatic TG levels and ameliorate disease progression [50]. Studies also indicate that genetic defects in apoB100 or MTTP directly lead to MASLD by impairing TG export [51, 52]. In summary, both apoB100 and MTTP represent critical determinants of hepatic lipid homeostasis in MASLD.

## 3 Cell Death

Hepatocellular death is a critical driver for the progression of ALD and MASLD to hepatitis and fibrosis. In ALD, ethanol induces different forms of programmed hepatocyte death through multiple signaling pathways, such as apoptosis, pyroptosis, and ferroptosis regulated by fibroblast growth factor 23 (FGF23), extracellular nicotinamide phosphoribosyltransferase (eNAMPT), gasdermin D (GSDMD), and Acyl‐coenzyme A synthetase short‐chain family member‐2 (ACSS2). In MASLD, multiple pathways, such as death receptors, autophagy‐related proteins, GSDMD, and hypoxia‐inducible factor 2α (HIF‐2α), are all involved in the initiation and progression of the disease. These mechanisms contribute to hepatic steatosis, inflammation, and fibrogenesis, thereby accelerating MASLD progression.

### 3.1 Cell Death in ALD

Apoptosis contributes to the progression of ALD. In ALD, ethanol drives hepatocyte autocrine release of FGF23 via the cannabinoid receptor 1–estrogen‐related receptor gamma–FGF23 signaling axis. This autocrine FGF23 subsequently activates the cytochrome P450 family 2 subfamily E member 1–ROS–mitochondrial apoptotic pathway, ultimately triggering hepatocyte death. This mechanism underscores the central role of FGF23 in OxS and apoptosis, providing a rationale for developing FGF23‐targeted therapeutic strategies for ALD [53].

Additionally, pyroptosis, a form of programmed inflammatory cell death, has been established in recent years as playing a crucial role in ALD development. Specifically, noncanonical caspase (CASP)‐11‐mediated cleavage of Gasdermin D (GSDMD) has been identified as a key mechanism mediating the progression from chronic ALD to alcoholic hepatitis (AH) [54]. Mechanistically, alcohol downregulates miR‐148a expression in hepatocytes via forkhead box protein O1, leading to thioredoxin‐interacting protein (TXNIP) overexpression and NOD‐like receptor protein 3 inflammasome activation. This activation recruits and activates CASP‐1, which subsequently cleaves the pyroptosis executioner protein GSDMD. Cleaved GSDMD forms pores in the plasma membrane and promotes the release of proinflammatory cytokines, such as IL‐1β and IL‐18, thereby inducing hepatocyte pyroptosis [55].

Emerging evidence has implicated ferroptosis and cuproptosis as critical mechanisms in the pathogenesis of ALD. Ethanol metabolism leads to acetate accumulation, which—upon suppression of ACSS2—disrupts histone acetylation‐dependent expression of hepcidin antimicrobial peptide 1/2, resulting in systemic iron dyshomeostasis and ferroptosis‐driven hepatic injury and inflammation [56]. Furthermore, ethanol stimulates extracellular secretion of eNAMPT from brown adipocytes. eNAMPT then induces hepatocyte ferroptosis via a Toll‐like receptor 4 (TLR4)‐dependent pathway involving mitochondrial ROS and ferritinophagy, thereby exacerbating ALD progression [57]. Additionally, downregulation of fibronectin type III domain‐containing protein 3B (FNDC3B) exacerbates ethanol‐induced steatosis and lipid peroxidation via AMPK inactivation and subsequent transferrin suppression, leading to iron overload and ferroptosis [58]. On the other hand, cuproptosis‐related genes (CRGs)—including DLAT, GLS, and CDKN2A—are upregulated in both human ALD samples and mouse models, and correlate negatively with alcohol‐metabolizing enzymes (ADH1B and ALDH2) [59]. These genes are involved in PI3K/AKT/mTOR signaling and immune pathways. Other CRGs (DPYD, SLC31A1, and DBT) also demonstrate diagnostic value and are linked to altered immune infiltration patterns in ALD [60]. Collectively, these findings underscore ferroptosis and cuproptosis as key pathways contributing to hepatic lipid metabolism dysfunction, injury, and inflammation in ALD. Targeting regulators of these cell death modes may offer novel therapeutic strategies.

### 3.2 Cell Death in MASLD

Cell death is an important process in the pathogenesis of MASLD. Apoptosis is a hallmark feature of MASLD [61]. In MASLD, chronic excessive lipid deposition induces ER stress, which upregulates the expression of proapoptotic genes and proteins, thereby triggering apoptosis. The extent of hepatocyte apoptosis positively correlates with the stages of inflammation and fibrosis [62]. Several studies have shown that multiple death receptors, such as tumor necrosis factor (TNF) receptor, Fas, and TNF‐related apoptosis‐inducing ligand receptor (TRAIL‐R), are upregulated in MASLD patients [63, 64, 65]. Activation of these receptors initiates intracellular cascades that activate CASP family members. Various MASLD animal models also exhibit elevated levels of CASP‐3/8/9 [62, 66, 67]. Similarly, selective knockdown of TRAIL or CASP‐8 effectively reduces hepatocyte apoptosis, suppresses inflammation and fibrosis, and prevents diet‐induced MASH [66, 68].

Dysfunctional autophagy is implicated in the pathogenesis of MASLD [69]. Autophagy is commonly downregulated in MASLD, likely due to impaired autophagosome–lysosome fusion, leading to the accumulation of undegraded autophagosomes and compromised lipid clearance. This process further promotes hepatic steatosis and hepatocyte apoptosis, thereby exacerbating MASLD progression [70, 71, 72]. Key autophagy‐related proteins such as Beclin1 (initiation of autophagy), microtubule‐associated protein 1 light chain 3 (LC3A) (autophagosome formation and elongation), and sequestosome 1 (SQSTM1)/p62 (a marker of impaired autophagy) play crucial roles in this process [73]. Hepatic lipid deposition downregulates the expression of Beclin1 and LC3A, thereby reducing autophagic activity and promotes lipid droplet accumulation in hepatocytes [74]. Furthermore, autophagy dysfunction leads to the aggregation of SQSTM1/p62, which not only indicates impaired autophagic flux but also contributes to the progression of MASLD [71, 75].

Pyroptosis accelerates the development of MASLD to irreversible states [76, 77]. GSDMD has been identified to play a key role in pyroptosis [78, 79, 80]. Evidence demonstrates that hepatocyte‐specific deficiency of nuclear receptor subfamily 5 group A member 2 (NR5A2) exacerbates MASH by inducing pyroptosis through downregulation of aldehyde dehydrogenase 1 family member B1 (ALDH1B1), leading to accumulated toxic aldehydes and OxS. This subsequently activates the CASP‐1–GSDMD pathway, triggering pyroptotic hepatocyte death [81]. Therefore, intervening in the NR5A2–ALDH1B1 axis has therapeutic potential in MASH. In summary, targeting these cell death mechanisms offers promising therapeutic strategies for attenuating MASLD and disease progression.

Ferroptosis and cuproptosis are involved in the progression of MASLD. In MASLD, hepatocyte‐specific HIF‐2α activation aggravates liver injury by inducing ferroptosis. Specifically, it upregulates divalent metal transporter 1 and suppresses ferroportin, leading to iron overload, lipid peroxidation, and glutathione depletion, and ultimately triggering ferroptosis and hepatocyte death. This mechanism provides a theoretical basis for targeting the HIF‐2α–iron axis as a therapeutic strategy for MASLD [82]. Furthermore, recent studies have linked “cuproptosis” to MASLD. Specifically, cuproptosis contributes to the progression of MASLD from simple steatosis to hepatitis and fibrosis by disrupting hepatic lipid metabolism and exacerbating OxS and inflammatory responses [83]. Although some evidence indicates that cuproptosis may influence MASLD pathogenesis through mechanisms such as modulating the hepatic immune microenvironment and inducing cell death, its precise role and underlying mechanisms remain incompletely elucidated [84, 85]. In conclusion, ferroptosis and cuproptosis are involved in the progression of MASLD by promoting the accumulation of metal ions, OxS, and inflammatory liver injury.

## 4 Cellular Senescence

In ALD, long‐term alcohol exposure can promote hepatocyte senescence. The key mechanisms include upregulation of ZNF281 leading to impaired PTEN‐induced kinase 1 (PINK1)/Parkin‐mediated mitophagy and dysregulation of the SIRT1–CCAAT/enhancer binding protein alpha (C/EBPalpha)–miR‐223 axis increasing susceptibility to ALD. In MASLD, senescent hepatocytes, on the one hand, propagate chronic inflammation and tissue damage through senescence‐associated secretory phenotype (SASP) and mitochondrial dysfunction; on the other hand, they modulate MASLD progression by promoting OxS through telomere shortening and p53/p21 dysregulation.

### 4.1 Cellular Senescence in ALD

In ALD, prolonged alcohol exposure increases OxS and inflammation, accelerating hepatocellular senescence and impairing liver function. Studies have shown that alcohol intake is associated with changes in the expression of ageing‐related genes, which may exacerbate the development of ALD [55, 86].

Aberrant upregulation of the transcription factor ZNF281 in senescent hepatocytes suppresses hexokinase 2 expression, which leads to impaired PINK1/Parkin‐mediated mitophagy, resulting in the accumulation of dysfunctional mitochondria and a further increase in ROS production, thereby promoting ALD progression [87]. The reduced expression or functional loss of certain ageing genes may also exacerbate ALD. Sirtuins or silent information regulators of gene transcription 1 (SIRT1) is an NAD^+^‐dependent deacetylase that modulates ageing‐associated genes like FoxO and p53 through deacetylation, affecting their activity [88]. SIRT1 promotes the transcriptional activation of miR‐223 by directly binding to the transcription factor C/EBPα and catalyzing its deacetylation. miR‐223 can subsequently suppress the IL‐6–p47phox–ROS axis and regulate neutrophil infiltration and activation [89]. Alcohol intake induces dual inhibitory effects on SIRT1, reducing both its expression and activity [90]. Ageing amplifies the dysregulation of the SIRT1–C/EBPα–miR‐223 axis, significantly increasing susceptibility to ALD.

### 4.2 Cellular Senescence in MASLD

Senescent cells secrete a group of cytokines with autocrine, paracrine, and endocrine activities, collectively termed SASP [91]. SASP factors IL‐8 and IL‐17 activate Kupffer cells (KCs), recruit neutrophils infiltrating the liver, and promote the formation of neutrophil extracellular traps (NETs), which release abundant ROS leading to hepatocyte lipid peroxidation [92]. In MASLD, senescent hepatocytes are characterized by mitochondrial damage, polyploidy, genomic instability and decreased regenerative capacity. They perturb hepatic homeostasis by secreting SASP and damaging mitochondrial β‐oxidation, promoting fat accumulation and disease progression [93, 94, 95, 96, 97].

Furthermore, telomere shortening and dysregulation of p53/p21 pathways exacerbate cellular aging [98]. As a transcription factor, p53 upregulates p21, a cyclin‐dependent kinase (CDK) inhibitor that, when overexpressed, inhibits CDK1, increasing OxS and accelerating MASLD [99]. Hepatocyte senescence exacerbates steatosis and disease progression of MASLD by promoting inflammatory responses, OxS and directly disrupting lipid metabolism.

## 5 Gut Dysbiosis

Numerous studies have revealed alterations in the gut microbiome in ALD and MASLD patients. Overall, the diversity of the gut microbiota in patients with ALD and MASLD is significantly reduced, including both bacteria and fungi. The gut microbiota and its metabolites play a crucial role in the onset and progression of ALD and MASLD [100, 101]. The metabolites related to ALD and MASLD include pathogen‐associated molecular patterns (PAMPs), short‐chain fatty acids (SCFAs), bile acids (BAs), and so on [102, 103].

### 5.1 Changes of the Microbiota in ALD

Numerous studies have revealed alterations in bacterial community structure in ALD patients (Table 1). As the severity of ALD increases, the gut microbiota undergoes pronounced compositional alterations accompanied by a progressive decline in phylogenetic diversity. Notably, *Fusobacterium* was observed to be elevated in patients with AH but decreased in those without, suggesting its potential association with specific disease stages [104, 105, 106, 107, 108]. Overall, these microbial community dysregulations are closely correlated with ALD progression, indicating that dysbiosis intensifies with advancing disease severity.

**TABLE 1:** Dynamic changes of the bacterial microbiome in ALD.

References	Participants	Method	Phylum	Genus (species)
Bajaj et al. [106]	Cirrhotic patients Etiologies other than solely alcohol (*n* = 170) vs. only alcohol‐related etiology (*n* = 43)	Multitagged pyrosequencing	Firmicutes Proteobacteria ↑	*Firmicutes_Clostridiales_ XIV* ↓ *Firmicutes_Lachnospiraceae* ↓ *Firmicutes_Ruminococcaceae* ↓ *Proteobacteria_Enterobacteriaceae* ↑ *Proteobacteria_Halomonadaeace* ↑
Yan et al. [107]	Alcohol‐treated group vs. control	16S rRNA gene sequencing	Firmicutes Verrucomicrobia ↑ Bacteroidetes ↓	*Lactococcus* ↓ *Pediococcus* ↓ *Lactobacillus* ↓ *Leuconostoc* ↓ *Bacteroidales* ↑ *Bacteroides* ↑ *Porphyromonadaceae* ↑
Fan et al. [105]	Adult male Wistar rats Alcohol dependence (*n* = 12) vs. alcohol withdrawal (*n* = 12)	High‐throughput 16S rRNA gene sequencing	Firmicutes Bacteroidetes ↓	*Bacilli* ↓ *Lactobacillales* ↓ *Christensenellaceae* ↑ *Ruminococcaceae* ↑ *Peptostreptococcaceae* ↑ *Butyricimonas* ↑ *Bacteroidales‐S24‐7* ↑ *Porphyromonadaceae* ↑
Smirnova et al. [109]	Alcohol‐related hepatitis patients (*n* = 34) vs. heavy drinking controls (*n* = 20)	16S rRNA gene sequencing	Firmicutes Bacteroidetes ↓ Proteobacteria ↑	*Coriobacteriaceae Atopobium* ↑ *Fusobacteriaceae Fusobacterium* ↑ *Coriobacteriaceae Eggerthella* ↓
Dubinkina et al. [104]	Alcohol dependence syndrome patients (*n* = 72) vs. alcohol‐related cirrhosis patients (*n* = 27)	Shotgun metagenomics	Firmicutes ↓	*Veillonella atypica* ↓ *Veillonella dispar* ↓ *Veillonella parvula* ↓
Dubinkina et al. [104]	Alcohol dependence syndrome patients (*n* = 72) vs. control (*n* = 60)	Shotgun metagenomics	Firmicutes ↑ Bacteroidetes ↓	*Klebsiella* ↑ *Lactococcus* ↑ *K. pneumoniae* ↑ *Lactobacillus salivarius* ↑ *Citrobacter koseri* ↑ *Lactococcus lactis subsp*. *cremoris* ↑ *Akkermansia* ↓
Dubinkina et al. [104]	Alcohol‐related cirrhosis patients (*n* = 27) vs. control (*n* = 60)	Shotgun metagenomics	Firmicutes ↑	*Bifidobacterium* ↑ (*B. longum, dentium, breve*) *Streptococcus* ↑ (*S. thermophilus, mutans*) *Lactobacillus species* ↑ (*L. salivarius, antri, crispatus*)
Ciocan et al. [108]	Cirrhotic with severe alcohol‐related hepatitis (*n* = 17) vs. cirrhotic without severe alcohol‐related hepatitis (*n* = 17)	16S rRNA gene sequencing	Firmicutes Actinobacteria ↑ Proteobacteria ↑ Enterobacteriaceae ↑ Bacteroidetes ↓	*Lactobacillus* ↑ *Lactococcus* ↑ *Actinomyces* ↑ *Bilophila* ↓ *Rothia* ↑ *Bifidobacterium* ↑ *Haemophilus* ↑ *Parabacteroides* ↓ *Oscillospira* ↓
Ganesan et al. [110]	Alcohol‐related fatty liver (*n* = 25), alcohol‐related hepatitis (*n* = 80), alcohol‐related cirrhosis (*n* = 80) vs. Control (*n* = 62)	16S rRNA sequencing and metabolite profiles	Bacteroidetes ↓ Proteobacteria ↑ Enterobacteriaceae ↑ Streptococcaceae ↑ Staphylococcaceae ↑ Enterococcaceae ↑ Lactobacillaceae ↑ Firmicutes ↑	*Eubacterium_g23* ↑ *Oscillibacter* ↑ *Dialister* ↑ *Paraprevotella* ↑ *Fusobacterium* ↑ *Bifidobacterium* ↑ *Haemophilus* ↑ *Staphylococcus* ↑ *Streptococcus* ↑ *Akkermansia* ↑ *Lactobacillus* ↑ *Prevotella* ↓ *Alistipes* ↓ *Bacteroides* ↓ *Parabacteroides* ↓ *Phascolarctobacterium* ↓ *Faecalibacterium* ↓

*Abbreviation*: 16S rRNA, 16S ribosomal RNA.

Although fungi constitute a minor fraction of the intestinal microbiota, several studies have recently highlighted the role of the mycobiome in the development and progression of ALD, partly due to the high mortality associated with invasive fungal infections [111]. Fungal alterations in ALD patients vary due to factors such as geographical location, dietary patterns, and cultural differences. However, nearly all relevant studies report an increase in *Candida albicans* abundance and a reduction in fungal diversity [111, 112, 113, 114, 115] (Table 2). Since fungi are commonly used in alcohol production, gut microbiome changes in ALD patients may be a consequence of alcohol intake, rather than directly linked to ALD. A balanced gut microbiome is crucial for maintaining host immune homeostasis. In addition, animal studies suggest that gavage with *Malassezia restricta* exacerbates ethanol‐induced liver damage by inducing inflammatory cytokines and chemokines in KCs, mediated by C‐type lectin domain family 4 member N signaling [116]. For example, in mice fed sake yeast, ethanol‐induced increases in TG and glutamate pyruvate transaminase levels were significantly attenuated, suggesting that sake yeast protects against alcohol‐related liver injury by maintaining methionine metabolism [117].

**TABLE 2:** Dynamic changes of the fungal microbiome in ALD.

References	Participants	Method	Genus	Species
Chu et al. [114]	Alcohol‐related hepatitis (*n* = 91), alcohol use disorder (*n* = 42) vs. controls (n = 11)	Quantitative PCR		*Candida albicans* ↑
Lang et al. [118]	Alcohol‐related hepatitis (*n* = 59), alcohol use disorder (*n* = 15) vs. controls (*n* = 11)	ITS Amplicon sequencing	*Candida* ↑ *Penicilllium* ↓ *Saccharomyces* ↓ *Debaryomyces* ↓	
Yang et al. [115]	Alcohol‐related cirrhosis (*n* = 4), alcohol‐related hepatitis (*n* = 6), alcohol use disorder (*n* = 10) vs. controls (*n* = 8)	ITS Amplicon sequencing	*Candida* ↑ *Epicoccum* ↓ *Debaryomyces* ↓	*Humicola* ↑ *Fusarium* ↑ *Aspergillus* ↑ *Candida parapsilosis* ↓ *Candida albicans* ↓

*Abbreviations*: ITS, internal transcribed spacer; PCR, polymerase chain reaction.

Alcohol consumption significantly impacts the gut virome composition, affecting both phage abundance and phage‐bacteria interactions. As ALD progresses, *Streptococcus* phages may exert lytic effects on their bacterial hosts. Furthermore, mammalian viruses, including members of the *Parvoviridae* and *Herpesviridae* families, were detected in fecal samples from patients with AH and showed a positive correlation with liver disease severity. Notably, increased viral diversity was associated with reduced bacterial diversity, a shift most pronounced in individuals with AH [119]. These findings highlight the crucial role of viral community disruption in the pathogenesis of AH and offer novel therapeutic opportunities through targeted modulation of the gut microbiota.

### 5.2 The Role of Gut Microbiota in the Progression of ALD

Although some microbiota differ in abundance and trends among patients, certain typical changes offer valuable diagnostic guidance. Studying changes in specific gut microbiota and their abundance in ALD patients helps differentiate ALD from other diseases with similar symptoms, such as viral and nonalcoholic liver diseases. This improves diagnostic accuracy and allows timely interventions to delay disease progression. For instance, studies have found that approximately 30% of ALD patients have cytolysin‐producing *Enterococcus faecalis* in their feces, a rate significantly higher than HC [120]. The presence of *Enterococcus faecalis* is strongly associated with higher mortality rates in patients [121]. Besides, kpsM‐positive *E. coli* is correlated with patient mortality and mouse model demonstrated that kpsM‐positive *E. coli* exacerbate ethanol‐induced liver disease [122]. This suggests that some microbiota such as *Enterococcus faecalis* and *E.coli* are directly implicated in ALD initiation and severity. Additionally, an increase in *Candida* has been detected in AH patients, and its exotoxin Candidalysin is positively correlated with the severity and mortality of alcohol‐related hepatitis [114]. Furthermore, certain specific microbial strains demonstrate diagnostic potential for distinguishing between ALD and MASLD. An analysis revealed a fungal signature—comprising *Scopulariopsis*, *Kluyveromyces*, *M. restricta*, and *Mucor*—that exhibited superior discriminatory performance in differentiating ALD from MASLD, achieving an area under the curve of 0.93. [123]

### 5.3 Changes in the Microbiota of MASLD

In patients with MASLD, significant changes occur in the structure of the intestinal microbiota compared with healthy individuals [124, 125]. For specific changes, please refer to the table below (Table 3). At the phylum level, the abundances of Gammaproteobacteria and Bacteroidetes increased [126, 127, 128]. The abundances of Firmicutes and Clostridia decreased [129, 130]. At the family level, the abundance of Lachnospiraceae, Ruminococcaceae, Lactobacillaceae, and Peptostreptococcaceae in MASLD decreased [125, 129, 131, 132, 133, 134]. The abundance of Lachnospiraceae and Enterobacteriaceae increased [124, 135]. Alterations in Lachnospiraceae remain controversial and warrant further investigation.

**TABLE 3:** Dynamic changes of the bacterial microbiome in MASLD.

References	Participants	Method	Genus	Species
Astbury et al. [125]	MASH patients (*n* = 55) vs. non‐MASH MASLD (*n* = 28)	16S rRNA sequencing	*Collinsella* ↑ *Blautia* ↓ *Lachnospiraceae* ↓ *Faecalibacterium* ↓	
Zhang et al. [136]	Mice: high‐fat/high‐cholesterol, normal chow diet/high‐fat/low‐cholesterol diet‐fed C57BL/6 (*n* = 8–19/group); human: hypercholesterolemia (*n* = 59) vs. healthy controls (*n* = 39)	16S rRNA sequencing; metagenomics	*Mucispirillum* ↑ *Desulfovibrio* ↑ *Anaerotruncus* ↑ *Helicobacter* ↑ *Clostridium* ↑ *Bifidobacterium* ↓ *Bacteroides* ↓ *Akkermansia* ↓ *Lactobacillus* ↓	*Mucispirillum schaedleri* ↑ *Helicobacter ganmani* ↑ *Clostridium celatum* ↑ *Clostridium cocleatum* ↑ *Clostridium methylpentosum* ↑ *Clostridium ruminantium* ↑ *Bacteroides acidifaciens* ↓ *Bacteroides eggerthii* ↓ *Bacteroides uniformis* ↓ *Akkermansia muciniphila* ↓
Behary et al. [128]	MASLD‐HCC patients (*n* = 60) vs. cirrhosis (*n* = 60) vs. healthy (*n* = 60)	Metagenomic sequencing	*Escherichia* ↑ *Streptococcus* ↑ *Klebsiella* ↑ *Akkermansia* ↓ *Coprococcus* ↓ *Bifidobacterium* ↓	*Escherichia coli* ↑ *Klebsiella pneumoniae* ↑ *Streptococcus vestibularis* ↑ *Streptococcus salivarius* ↑ *Akkermansia muciniphila* ↓
Oh et al. [130]	Cirrhosis patients (*n* = 163) vs. noncirrhosis (*n* = 134)	Metagenomic sequencing	*Veillonella* ↑ *Streptococcus* ↑ *Enterococcus* ↑ *Rothia* ↑ *Clostridium* ↑ *Bacteroides* ↓	*Veillonella parvula* ↑ *Streptococcus salivarius* ↑ *Enterococcus faecium* ↑ *Clostridium bolteae* ↑
Monga Kravetz et al. [137]	Obese youth with MASLD (*n* = 64) vs. obese without MASLD (*n* = 33)	16S rRNA sequencing	*Bacteroides* ↓ *Ruminococcus* ↑ *Dorea* ↑ *Blautia* ↑	
Iino et al. [133]	MASLD patients (*n* = 201) vs. healthy controls (*n* = 201)	16S rRNA sequencing	*Faecalibacterium* ↓ *Bacteroides* ↑	
Yang et al. [138]	MASLD patients (*n* = 30) vs. healthy controls (*n* = 30)	16S rRNA sequencing	*Bacteroides* ↑ *Escherichia‐Shigella* ↑ *Blautia* ↑ *Faecalibacterium* ↓ *Ruminococcus* ↓ *Eubacterium* ↓	
Nistal et al. [139]	Obese MASLD patients (*n* = 57) vs. obese controls (*n* = 20)	16S rRNA sequencing	*Bacteroides* ↓ *Prevotella* ↓ *Faecalibacterium* ↓ *Oscillospira* ↓ *Ruminococcus ↓* *Blautia* ↑	
Caussy et al. [140]	MASLD‐cirrhosis patients (*n* = 163) vs. MASLD noncirrhosis (*n* = 134)	Metagenomic sequencing	*Veillonella* ↑ *Streptococcus* ↑ *Enterococcus* ↑ *Rothia* ↑ *Clostridium* ↑ *Escherichia* ↑ *Klebsiella* ↑ *Bacteroides* ↓	*Veillonella parvula* ↑ *Streptococcus salivarius* ↑ *Streptococcus anginosus* ↑ *Enterococcus faecium* ↑ *Escherichia coli* ↑ *Klebsiella pneumoniae* ↑
Yang et al. [141]	MASLD patients (*n* = 60) vs. healthy controls (*n* = 30)	16S rRNA sequencing	*Bacteroides* ↑ *Escherichia‐Shigella* ↑ *Streptococcus* ↑ *Blautia* ↑ *Faecalibacterium* ↓ *Ruminococcus* ↓ *Eubacterium* ↓	
Lanthier et al. [142]	Obese patients with steatosis/fibrosis (*n* = 74) vs. obese without (*n* = 27)	16S rRNA sequencing	*Ruminococcus* ↑ (with fibrosis) *Blautia* ↓ *Faecalibacterium* ↓ *Subdoligranulum* ↓	

*Abbreviations*: 16S rRNA, 16S ribosomal RNA; HCC, hepatocellular carcinoma; MASH, metabolic dysfunction‐associated steatohepatitis; MASLD, metabolic dysfunction‐associated steatotic liver disease.

The changes in the fungal community are detailed in the table below (Table 4). Compared with healthy subjects, the relative abundance of Talaromyces, Paraphaeosphaeria, Lycoperdon, Curvularia, Phialemoniopsis, Paraboeremia, Sarcinomyces, Cladophialophora, and Sordaria was increased in patients with MASLD while the abundance of Leptosphaeria, Pseudopithomyces, and Fusicolla was decreased [143]. Patients with MASH or advanced fibrosis had a distinct fecal mycobiome as compared with simple steatosis or minimal fibrosis, which was characterized by increased C. albicans, Babjeviella inositovora, Mucor sp., unknown Hanseniaspora, unknown Pleosporales, and Pichia barkeri [144]. MASLD severity were associated with reduced viral diversity and a lower proportion of bacteriophages (phages). Compared with other enteroviruses, specific gut viral taxa, such as Lactococcus phages, were decreased in patients with advanced fibrosis [145].

**TABLE 4:** Dynamic changes of the fungal microbiome in MASLD.

References	Participants	Method	Genus	Species
Niu et al. [146]	MASLD patients (*n* = 32) vs. healthy controls (*n* = 31)	ITS2 sequencing	*Saccharomyces* ↑ *Candida* ↑ *Malassezia* ↑ *Aspergillus* ↑	
You et al. [143]	MASLD patients (*n* = 73) vs. healthy controls (*n* = 73)	ITS2 sequencing	*Candida* ↑ *Saccharomyces* ↑ *Cladosporium* ↑ *Cyberlindnera* ↑ *Aspergillus* ↑	
Demir et al. [144]	MASLD patients (*n* = 78) vs. healthy controls (*n* = 105)	ITS sequencing; fungal culture	*Candida* ↑ *Saccharomyces* ↑ *Malassezia* ↑ *Rhodotorula* ↑	*Candida albicans ↑* *Candida krusei ↑* *Saccharomyces cerevisiae ↑* *Malassezia restricta ↑* *Malassezia globosa ↑*

*Abbreviations*: ITS, internal transcribed spacer; MASLD, metabolic dysfunction‐associated steatotic liver disease.

### 5.4 The Relationship Between Microbiota and the Progression of MASLD

The assessment of MASLD progression risk utilizing metabolite and gut microbiota markers has been explored [147]. Mendelian randomization analyses indicate that Trichosporonaceae UCG‐004, Enterobacteriaceae, Prevotellag, and Enterobacteriales are associated with an elevated risk of MASLD. An increased risk of MASH is linked to Veillonella and Dorea, whereas Oscillospira and Ruminococcaceae UCG‐013 are connected to a reduced risk [148]. Furthermore, class Mollicutes, class Deltaproteobacteria, and phylum Tenericutes demonstrated significant associations with MASLD risk [149]. Robust discrimination between MASLD patients and healthy controls can be achieved using the bacterial genera Faecalibacterium, Subdoligranulum, Haemophilus, and Roseburia, in addition to butyric and acetic acids [150]. Negative correlations with MASLD were identified for Lactobacillaceae, Intestinibacter, and Christensenellaceae through a two‐sample Mendelian randomization analysis, while positive correlations were observed for Coriobacteriia, Oxalobacteraceae, Actinomycetales, and Ruminococcaceae_UCG005 [151]. A recent investigation confirmed a causal relationship linking the progression of MASLD to the abundance of eight gut microbial taxa [152]. Positive associations were noted for the order Actinomycetales, NB1n, the family Oxalobacteraceae, Ruminococcaceae, and Actinomycetaceae; negative associations were found for the Lactobacillaceae, Intestinibacter, and Christensenellaceae R7 group regarding MASLD onset. A novel study further reports that steatosis severity in MASLD correlates strongly with gut microbial dysbiosis and altered community composition [153]. Dysregulation of the enteric virome—particularly bacteriophages—also emerges as a novel and clinically relevant indicator of MASLD severity, offering new virological perspectives on disease pathogenesis [145].

### 5.5 Mechanism of Microbiota Involved in the Progression of ALD and MASLD

Dysfunction of the gut–liver axis constitutes a central mechanism in the pathogenesis of ALD and MASLD [154, 155]. Alcohol disrupts intestinal barrier integrity, facilitating the translocation of microbiota‐derived molecules, such as LPS, into the portal circulation. This translocation activates hepatic immune cells, representing a critical step in driving liver inflammation. Overactivation of the immune system affects liver lipid metabolism and OxS status through a series of complex mechanisms, thereby promoting the progression of MASLD. Furthermore, metabolites derived from the gut microbiota, including SCFAs and BAs, critically modulate the course of ALD and MASLD through host receptor‐mediated pathways, illustrating the multifaceted role of the microbiome in steatotic liver diseases.

#### 5.5.1 Metabolites and Toxins

Gut microbiota metabolites (such as SCFAs, BAs, and tryptophan derivatives) and bacterial/fungal components (such as cytolysi, candidalysin, and β‐glucan) profoundly influence the disease progression of ALD and MASLD by regulating key receptors and signaling pathways such as farnesoid X receptor (FXR), AMPK, and PPARα.

SCFAs, such as acetate, propionate, and butyrate, are metabolic end products generated by gut bacterial fermentation of indigestible dietary fiber. These SCFAs activate G protein‐coupled receptors GPR41 and GPR43 on intestinal epithelial cells, leading to mitogen‐activated protein kinase (MAPK) signaling and the rapid production of chemokines and cytokines, which orchestrate protective immunity and tissue inflammation in mice [156, 157]. In the context of ALD, butyrate and other SCFAs ameliorate liver injury by suppressing M1 macrophage polarization and promoting a shift toward anti‐inflammatory M2 macrophages, thereby reducing inflammation [158]. In MASLD, patients exhibit elevated total SCFA levels [159]. SCFAs exert protective effects by enhancing PPARα expression, activating AMPK signaling to enhance fatty acid oxidation, and suppressing lipogenesis via SREBP1c downregulation [160, 161, 162]. Butyric acid, in particular, plays a key role in MASLD through several mechanisms: counteracting intestinal permeability and bacterial/LPS translocation [101], reducing inflammation and fat deposition via microbiota and barrier modulation [163], and reshaping microbial composition to suppress endotoxins and proinflammatory cytokines [164].

BA metabolism exhibits substantial alterations in ALD. Alcohol consumption upregulates key enzymes involved in BA metabolism, including Cyp7A1, Cyp27a1, Cyp8b1, and Baat, as well as transporters such as Bsep, Mrp2, P‐gp, and Asbt, while simultaneously suppressing expression of the hepatic/ileal transporter Ntcp and the nuclear receptor FXR [165]. Alcohol feeding also suppresses intestinal FXR transcription and increases miR‐194 expression. Mechanistically, this effect is mediated by the long noncoding RNA taurine upregulated gene 1 (Tug1) regulating taurine metabolism via the gut microbiota. Oral lithocholic acid‐3‐sulfate enriches bile salt hydrolase (BSH)‐active bacteria, which raises taurine levels and upregulates Tug1. This inhibits miR‐194, activates FXR–FGF15/19 signaling, and reduces hepatic BA synthesis and lipogenesis, thereby ameliorating ALD [166]. Patients with MASLD also exhibit abnormal BA metabolism. In MASLD, gut dysbiosis alters the BA pool composition, characterized by shifts in secondary BA ratios and suppressed FXR signaling [167]. Mechanistically, reduced abundance of BSH active bacteria impairs deconjugation of primary BAs, further inhibiting FXR activation. Decreased FXR activity attenuates suppression of SREBP1c‐mediated lipogenesis and dysregulates CYP7A1 expression, collectively promoting hepatic lipid accumulation, inflammation, and MASLD progression [168]. CYP7A1 is the rate limiting enzyme of the classical BA synthesis pathway. A Bruton's tyrosine kinase inhibitor  reduces BA synthesis by activating FXR and downregulating CYP7A1 expression, thereby ameliorating hepatic lipid metabolism and inflammation in MASLD [169]. The above indicates that the gut microbiota also profoundly influences BA metabolism and transformation. The interaction between disordered BA metabolism and the gut microbiota further exacerbates metabolic disorders in patients with MASLD [168, 170].

The gut microbiota metabolizes dietary tryptophan into indole and its derivatives, such as indole‐3‐acetic acid and indole‐3‐propionic acid (IPA), which are crucial for maintaining intestinal mucosa homeostasis [171]. Indole metabolism is intricately linked to ALD pathways, as IPA inhibits hepatic nuclear factor‐κB (NF‐κB) signaling, reducing liver inflammation and damage [172]. A study showed that oral administration of IPA significantly improves the gut environment in mice [173]. In ALD patients, a significant decrease in indole and its derivatives was found, potentially explaining liver injury [110]. Similarly, indole and its derivatives are also mechanistically implicated in MASLD progression. Previous studies have shown that in the diet‐induced MASLD phenotype of mice, indole supplementation can effectively alleviate liver lipid accumulation and inflammatory cell infiltration and reduce obesity and insulin resistance [174]. Other studies have shown that IPA can repair the intestinal barrier, reduce the entry of endotoxins into the bloodstream, and inhibit the TLR4/NF‐κB inflammatory signaling pathway, thereby effectively improving MASH in rats [172].

In ALD, bacterial/fungal components such as cytolysi, β‐glucan, and candidalysin serve critical roles in contributing to hepatocellular injury [119, 120]. As a major fungal cell wall polysaccharide, β‐glucan translocates into systemic circulation due to alcohol‐induced gut fungal overgrowth and barrier disruption. It binds to C‐type lectin domain containing 7A (CLEC7A) on KCs, activates the NLRP3 inflammasome and CASP‐1, and promotes IL‐1β secretion, contributing to hepatocyte damage and hepatic inflammation in ALD mouse model [115]. Meanwhile, *Candida albicans*‐derived candidalysin is upregulated in ALD and correlates with disease severity. It disrupts intestinal epithelial integrity and promotes the recruitment of immune cells. In the liver, candidalysin enhances the Th17 response and stimulates KCs via IL‐17 signaling, leading to increased production of inflammatory cytokines such as IL‐1β, CXCL1, and CXCL2, which exacerbate alcohol‐induced liver injury [118, 175]. Notably, β‐glucan may exhibit context‐dependent effects, with some studies suggest a protective role in acute liver injury through alternative pathways such as Adam‐17 activation [176]. Although there are relatively few reports about candidalysin in MASLD, existing studies have shown that candidalysin may activate the NLRP3 inflammasome, which plays a significant role in the development of liver inflammation and fibrosis in MASH [177, 178]. The dual effect of β‐glucan is also reflected in MASLD. On the one hand, β‐glucan binds to CLEC7A to induce liver inflammation. However, CLEC7A significantly increases in the livers of MASH patients and mice on a HFD, exerting harmful effects by inducing inflammatory factors such as transforming growth factor β1 (TGF‐β1) [179]. On the other hand, Many studies, such as yeast β‐glucan, *Aureobasidium pullulans*‐derived β 1,3–1,6 glucans, and (1–3)‐β‐d‐glucan isolated from traditional Chinese medicine, have demonstrated their antifibrotic, anti‐inflammatory, liver‐protecting, and lipid metabolism‐regulating effects [180, 181, 182]. However, the protective mechanism of β‐glucan in metabolic diseases remains unclear. In conclusion, in ALD and MASLD, both molecules contribute significantly to inflammation and hepatocellular damage, highlighting the complex role of fungal mediators in disease progression.

#### 5.5.2 Immunity and Receptor Regulation

Dysbiosis of the intestinal microbiota induces inflammatory responses and lipid metabolism disorders by disrupting the intestinal barrier and activating immune signaling pathways such as TLR4/NF‐κB in the liver. Meanwhile, dysbiosis of pathways such as BA receptor FXR further exacerbates the disease process.

Altered intestinal microbiota induces hepatic injury and barrier dysfunction, leading to ROS generation. This oxidative and ER stress, along with fatty acid accumulation, causes hepatic steatosis [183]. Increased intestinal permeability allows substances like LPS to enter the bloodstream, activating KCs and the NF‐κB pathway. This activation involves the kinase complex IKK, phosphorylation, and degradation of the NF‐κB inhibitor IκBα, leading to the release of NF‐κB transcription factors that trigger the expression of inflammation‐related genes, such as TNF‐α [184]. These processes exacerbate liver inflammation and injury. We further demonstrate that chronic ethanol exposure expands intestinal *Candida albicans*‐primed Th17 cells, which subsequently exacerbate alcohol‐related liver injury [175]. Neutrophils, first‐line granulocytes recruited during inflammation, mediate parenchymal cell damage, while macrophages clear pathogens and debris [185]. In addition, microbiota‐induced immune dysregulation may also trigger sterile hepatic inflammation via excessive immune responses [11].

Immune and receptor regulation critically regulate MASLD progression. Due to increased gut permeability, microbial‐derived products such as LPS reach the liver, where elevated serum levels correlate with disease severity and promote inflammation via TLR4‐mediated activation of NF‐κB and MAPK signaling, leading to the production of proinflammatory cytokines (e.g., TNF‐α, IL‐6) and chemokines (e.g., interferon‐γ [IFN‐γ] and monocyte chemoattractant protein‐1) [186, 187]. These gut‐derived PAMPs are recognized by pattern recognition receptors (e.g., TLRs, inflammasomes), with TLR4/NF‐κB signaling exacerbating inflammation, steatosis, and fibrosis through Jagged1 upregulation [188, 189]. PAMPs in MASLD mice promote M1 macrophage polarization via TLR2–NF‐κB/NLRP3–CASP‐1 and reprogram lipid metabolism toward lipogenesis through mTOR‐ribosomal protein S6 kinase beta‐1–SREBP‐1 signaling [190]. Collectively, these mechanisms highlight how the gut microbiota, through its structural components (PAMPs), orchestrates a complex network of immune and receptor‐mediated pathways that fundamentally drive MASLD pathogenesis.

## 6 Immunomodulation

**FIGURE 2:**
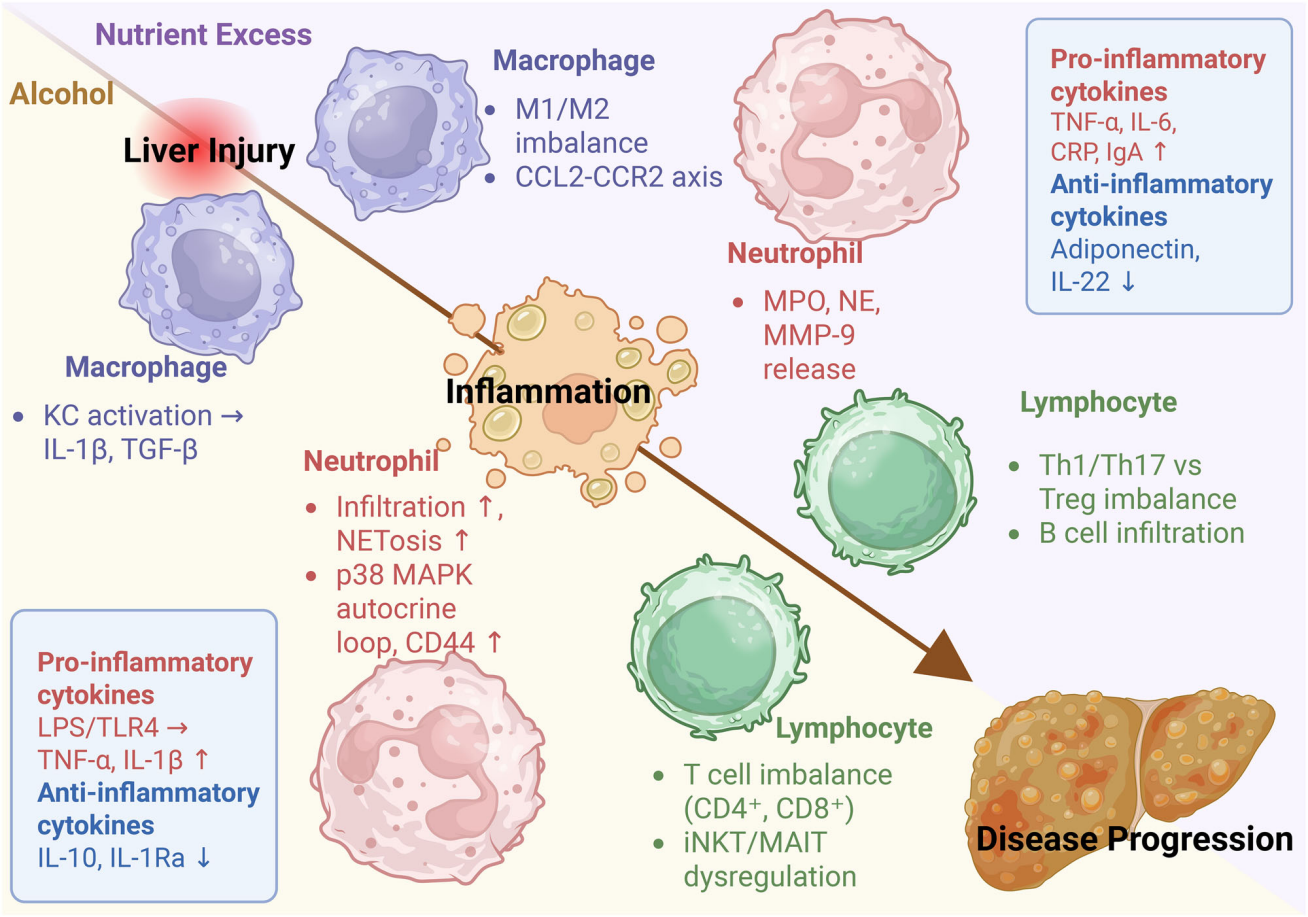
Comparative overview of immune dysregulation in ALD and MASLD. A convergent pathway of hepatic immune dysregulation drives the progression of both ALD and MASLD from initial injury to sustained inflammation and fibrosis. Despite differing etiologies (ethanol metabolism vs. nutritional excess/insulin resistance), both diseases feature hyperactivation of innate and adaptive immune responses. In ALD, gut‐derived PAMPs (e.g., LPS) and DAMPs activate Kupffer cells (KCs), leading to proinflammatory cytokine release (IL‐1β, TGF‐β). Neutrophil infiltration is a hallmark, exacerbated by a p38 MAPK‐dependent autocrine loop in IL‐8+ subsets and enhanced by surface CD44 expression. Adaptive immunity is dysregulated, characterized by intrahepatic accumulation of activated CD4+ and CD8+ T cells, loss of intestinal CD8+ TRM cells, and altered function of iNKT/MAIT cells. A pervasive cytokine imbalance features upregulation of TNF‐α, IL‐1β, and IFN‐γ, alongside suppression of anti‐inflammatory mediators like IL‐10 and IL‐1Ra. In MASLD, metabolic stress (e.g., fatty acids, oxidative stress) is the primary trigger. Hepatic macrophages are activated via TLR4/NF‐κB and CCL2–CCR2 signaling, promoting a proinflammatory M1 phenotype. Neutrophils drive injury via MPO, NE, and MMP‐9, which induce oxidative stress, hepatocyte death, and HSC activation. The adaptive immune response is skewed toward a proinflammatory state, with an increased Th17/Treg cell ratio and B cell infiltration contributing to disease activity. The cytokine profile is characterized by elevated proinflammatory factors (TNF‐α, IL‐6, CRP, IgA) and a deficiency in protective factors (adiponectin, IL‐22). *Abbreviations*: ALD, alcoholic liver disease; CCL2, C‐C motif chemokine ligand 2; CRP, C‐reactive protein; DAMPs, damage‐associated molecular patterns; HSCs, hepatic stellate cells; IgA, immunoglobulin A; IL, interleukin; iNKT, invariant natural killer T cells; KCs, Kupffer cells; LPS, lipopolysaccharide; MAPK, mitogen‐activated protein kinase; MASLD, metabolic dysfunction‐associated steatotic liver disease; MMP‐9, matrix metalloproteinase‐9; MPO, myeloperoxidase; NE, neutrophil elastase; NETs, neutrophil extracellular traps; PAMPs, pathogen‐associated molecular patterns; TGF‐β, transforming growth factor beta; Th, T helper cell; TLR4, Toll‐like receptor 4; TNF‐α, tumor necrosis factor alpha; Treg cell, regulatory T cell.

The pathological progression of ALD and MASLD is intimately linked to persistent and maladaptive hepatic immune responses. A comprehensive understanding of this process necessitates examining the distinct roles of immune cells and immune molecules (Figure 2).

### 6.1 Immune Cells

The immune cells that orchestrate the progression of ALD and MASLD mainly include macrophages, neutrophils, and T/B lymphocytes—which collectively drive steatosis, inflammation, and fibrosis. In ALD, hepatic macrophages, particularly KCs, are activated by damage‐associated molecular patterns (DAMPs) released from injured hepatocytes and PAMPs such as LPS. These stimuli promote a proinflammatory phenotype, characterized by the secretion of cytokines such as IL‐1β and TGF‐β [191]. KC‐derived extracellular vesicles carrying IL‐1β contribute to steatosis, while TGF‐β and 2‐arachidonoylglycerol promote fibrogenic activation of hepatic stellate cells (HSCs) and lipid accumulation [192]. Neutrophils play a pivotal role as key effector cells in the acute inflammatory response of ALD. They infiltrate the liver in response to chemotactic signals and exacerbate injury through the release of ROS, proteases, and NETs, which promote hepatocyte apoptosis and amplify inflammatory responses [191]. A recently identified subset of IL‐8+ neutrophils in sAH perpetuates its own activation and recruitment via a p38 MAPK‐dependent autocrine loop, leading to uncontrolled inflammation and disease progression [193]. Furthermore, alcohol exposure upregulates the expression of CD44 glycoprotein on neutrophil surfaces, enhancing their hepatic recruitment and activation [194]. Once activated, neutrophils release a broad spectrum of inflammatory mediators that aggravate hepatocellular damage and sustain the inflammatory cascade.

Lymphocyte subsets exhibit profound alterations in ALD, playing key roles in both immunoregulation and disease pathogenesis. In the liver, CD4+ T cells accumulate substantially, with a distinct terminally differentiated subpopulation—characterized by high GZMK expression and tissue‐resident features—enriched in fibrotic areas, where it may contribute to disease progression [195]. Concurrently, CD8+ T cells also increase substantially intrahepatically and exhibit upregulation of survival and activation genes such as Bcl2, further exacerbating alcohol‐induced liver injury [196]. Within the intestine, alcohol exposure leads to a significant reduction and enhanced apoptosis of CD8+ tissue‐resident memory T (TRM) cells in the duodenum. Their loss is strongly associated with impaired intestinal barrier function, bacterial translocation, and exacerbated hepatic inflammation [196, 197]. Furthermore, unconventional T cells—including invariant natural killer T (iNKT) and mucosal‐associated invariant T (MAIT) cells—exhibit altered frequencies and dysregulated cytokine profiles (e.g., elevated IFN‐γ, TNF‐α, and IL‐17A) in both ALD patients and experimental models, thereby promoting hepatic inflammation and disease advancement [198]. Additionally, an imbalance between regulatory T (Treg) cells and Th1 cells has been implicated in ALD‐related immune dysregulation, suggesting that restoring this balance could represent a promising therapeutic strategy [199]. Furthermore, the gut‐associated lymphoid tissue (GALT) plays a crucial role in maintaining intestinal barrier integrity and immune homeostasis. Alcohol‐induced impairment of GALT function reduces immune cell numbers and compromises pathogen recognition, facilitating the translocation of microbial products into the portal circulation and driving hepatic inflammation [200, 201].

Hepatic macrophage activation is an early and critical event in MASLD pathogenesis [202, 203]. These cells polarize into proinflammatory M1 or anti‐inflammatory M2 phenotypes based on microenvironmental cues. Hepatic macrophages mainly include KCs and monocyte‐derived macrophages (MoMφs) [204]. KCs activate via LPS, fatty acids, and OxS, differentiating into M1 phenotypes through TLR4/myeloid differentiation primary response 88/NF‐κB signaling to secrete TNF‐α, IL‐6, IL‐8, and inducible nitric oxide synthase (iNOS), exacerbating MASLD. MoMφs influence M1/M2 balance via C‐C motif ligand 2 (CCL2)‐C‐C motif chemokine receptor 2 (CCR2) signaling [205]. During the repair stage of MASLD and MASH, M2 type can replace M1 type, suggesting therapeutic potential in modulating macrophage polarization [206]. Neutrophils drive MASH progression [207, 208] by releasing myeloperoxidase (MPO), neutrophil elastase (NE), and matrix metalloproteinase‐9 (MMP‐9), which amplify OxS and liver injury [209, 210, 211, 212]. MPO induces mitochondrial permeability transition pore formation, hepatocyte death [213], and DNA damage, accelerating MASLD progression toward malignancy [214]. NE deficiency attenuates liver inflammation [215], while MPO/MMP‐9 activate HSCs, promoting fibrosis [216].

B and T lymphocytes regulate MASLD/MASH adaptive immunity [213, 217, 218]. B‐cell infiltration correlates with MASLD activity scores [219], partially mediated through T‐cell interactions. T‐cell subsets include CD4+, CD8+, and γδ T cells, with CD4+ T cells further classified into Th1, Th2, Th17, and Treg cells. Th1 and Th17 cells promote MASLD via IFN‐γ and IL‐17 production [220]. MASLD patients exhibit elevated peripheral Th1 levels, with hepatic lipotoxicity driving naive T‐cell differentiation into Th1 cells [221]. In addition, Treg cells are also regarded as playing a pivotal role in the progression of MASLD [222]. An increased Th17/Treg cell ratio in blood and liver marks MASLD to MASH progression [223].

In summary, ALD and MASLD progression are driven by a complex crosstalk among innate and adaptive immune cells. Macrophages and neutrophils initiate and amplify inflammation and fibrosis, while T and B lymphocytes modulate adaptive immune responses.

### 6.2 Immune Molecules

In ALD, the expression of multiple proinflammatory cytokines is significantly upregulated, collectively driving disease progression. Research has revealed that hepatocytes under alcohol‐induced stress secrete leukocyte cell‐derived chemotaxin 2 (LECT2). LECT2 binds to prohibitin 2 (PHB2) on neutrophil membranes, disrupting the stability of the PHB1/PHB2 heterodimer complex. This disruption leads to ROS accumulation, which in turn potentiates the formation of NETs, ultimately exacerbating liver injury [224]. Simultaneously, alcohol‐induced intestinal barrier dysfunction leads to elevated LPS levels, which activate TLR4 receptors on hepatic immune cells and trigger a potent innate immune response. This promotes the robust release of proinflammatory cytokines such as TNF‐α and IL‐1β, further amplifying hepatic inflammation [225, 226]. Together, these proinflammatory mediators form a positive feedback loop that sustains immune activation, hepatocyte death, and fibrogenesis.

In contrast, the expression of multiple anti‐inflammatory factors is suppressed in ALD, impairing the body's immunoregulatory capacity. Studies have shown that key anti‐inflammatory cytokines such as IL‐10 and IL‐4 are significantly reduced in the serum of ALD patients, while proinflammatory mediators including TNF‐α, IL‐1β, IFN‐γ, and IL‐6 are markedly elevated. This imbalance is associated with CD14 gene polymorphisms (e.g., the rs2569190 TT genotype), which enhance CD14 expression and TLR4 signaling, thereby exacerbating inflammatory responses [227]. Furthermore, functional deficiency of IL‐1 receptor antagonist (IL‐1Ra)—a critical negative regulator of IL‐1 signaling—leads to hyperactivation of IL‐1β pathways. This promotes CASP‐1‐dependent inflammasome activation, contributing to hepatic steatosis, inflammation, and injury [228]. These findings suggest that impaired production and function of anti‐inflammatory factors allow uncontrolled proinflammatory responses to persist and worsen, ultimately driving the progression toward hepatitis and fibrosis.

During the progression of MASLD, the balance of immune molecules (including cytokines and immunoglobulins) is disrupted, manifested as continuous upregulation of proinflammatory factors, and impaired function of anti‐inflammatory factors, jointly driving liver inflammation, fibrosis, and metabolic disorders. Canonical proinflammatory factors include TNF‐α, IL‐6 and C‐reactive protein (CRP). TNF‐α is a main factor leading to the onset and prognosis of MASLD, and its level is significantly increased in patients [229]. Similarly, IL‐6 expression is elevated in the liver and serum of MASH patients, where it is closely associated with the degree of liver inflammation and fibrosis [230]. A key mechanism of IL‐6 is its induction of the acute‐phase protein CRP. High‐sensitivity CRP (hs‐CRP) is persistently and significantly elevated in MASH and is inextricably linked to the severity of liver fibrosis, serving as a reliable biomarker of systemic inflammation [231]. Furthermore, beyond traditional cytokines, elevated serum IgA levels have been identified in MASLD patients and identified as an independent predictor of advanced fibrosis [232, 233, 234]. A recent mechanistic study has provided evidence that IgA promotes the initiation of liver fibrosis by driving the activation of MoMφs in the liver [235].

In terms of anti‐inflammatory factors, adiponectin, a crucial adipokine, exerts multiple protective effects in MASLD, including anti‐inflammatory actions, enhanced fatty acid oxidation, and improved insulin sensitivity [236, 237]. However, its levels are downregulated in MASLD, and studies have indicated a direct positive correlation between reduced adiponectin and MASLD‐related liver injury [238, 239]. For example, one study demonstrated that adiponectin exert protective effects against MASH by modulating the AMPK–c‐Jun N‐terminal kinase/extracellular regulated protein kinases 1/2–NF‐κB/ROS signaling pathway, thereby suppressing NLRP3 inflammasome activation and reducing the release of proinflammatory cytokines such as IL‐1β and IL‐18 [240]. Other typical anti‐inflammatory cytokines include IL‐22 and IL‐10 [229, 241]. The hepatoprotective effects of IL‐22 are likely mediated through inhibition of NLRP3 inflammasome activation, attenuation of local hepatic inflammation, suppression of HSC activation and fibrogenic activity, and/or induction of senescence in activated HSCs, collectively inhibiting the progression of liver fibrosis [242, 243]. Furthermore, targeting IL‐22 has shown promising therapeutic efficacy in MASLD [244, 245]. Although IL‐10 is known for its anti‐inflammatory and antifibrotic benefits, evidence supporting its specific role in MASLD remains relatively limited [246, 247].

Liver immune disorder is the core mechanism of ALD and MASLD. It is manifested as excessive activation of innate and adaptive immunity. Multiple immune cells, immune factors, and cell molecules jointly promote the progression of diseases.

## 7 Therapeutics

For ALD, sustained alcohol abstinence and nutritional support remain the mainstay of management. However, hepatic inflammation and fibrosis can persist even after cessation of alcohol consumption, and severe cases may only attain survival benefit through liver transplantation. Furthermore, the pathogenesis of ALD is multifaceted. Building upon the deep mechanistic understanding outlined above, therapeutic strategies for ALD are evolving from traditional supportive care toward mechanism‐based targeted therapies. Lifestyle modification remains the cornerstone of MASLD management, while metabolic modulation and gut microbiota‐targeted therapies are emerging as key treatment strategies [248].

### 7.1 Targeting Lipid Metabolism

The metabolic reprogramming of hepatic lipid pathways—encompassing DNL, β‐oxidation, and lipid export—serves as a central driver of liver injury in both ALD and MASLD. Recent efforts to therapeutically modulate these pathways have demonstrated encouraging preclinical and clinical results.

Aberrant hepatic lipid metabolism constitutes an early hallmark of ALD, providing a therapeutic avenue. Emerging studies highlight enhanced fatty acid oxidation and suppression of DNL as promising therapeutic strategies for ALD. Overexpression of TBC1D15 attenuates alcohol‐induced steatosis by modulating lipid droplet–mitochondria interplay, which reverses transcriptional upregulation of lipogenic genes and repression of lipolytic genes, thereby reducing hepatic lipid accumulation. Concurrently, TBC1D15 preserves mitochondrial integrity and activates genes central to fatty acid β‐oxidation [38]. Similarly, soluble dietary fibers such as inulin attenuate liver injury through microbial fermentation‐derived propionate, which activates PPARα and enhances fatty acid oxidation, mitigating lipid accumulation. Additionally, fiber‐mediated modulation of the gut–microbiota–BA axis reinforces intestinal barrier integrity and attenuates hepatic inflammation [249]. Further, alcohol‐induced activation of serine–arginine‐rich protein kinase 2 (SRPK2) in hepatocytes promotes lipogenesis via SREBP1 in ALD. Notably, FGF21 promotes SRPK2 degradation through mTORC1 inhibition, supporting targeted inhibition of the SRPK2 pathway as a potential therapeutic strategy [250].

In addition to targeting lipid synthesis, several therapeutic approaches also focus on other aspects of lipid metabolism. In a separate study, RNF2 was found to influence hepatic lipid metabolism through direct interaction with USP7 and modulation of the PI3K/AKT signaling pathway. Notably, RNF2 knockout effectively ameliorates ethanol‐mediated lipid accumulation and inflammatory status [30]. In drug discovery, an antialcoholic fatty liver disease (AFLD) polysaccharide, APFC‐2, isolated and purified from *Cornus officinalis* (Asiatic dogwood), has been demonstrated to significantly ameliorate alcohol‐induced hepatic steatosis in both HepG2 cells and AFLD mouse livers by modulating the liver kinase B1 (LKB1)/AMPK signaling pathway [251]. This identifies APFC‐2 as a potential therapeutic candidate for AFLD.

PPAR‐targeted therapies have shown therapeutic potential for MASLD treatment. Saroglitazar, a PPARα/γ dual agonist, significantly reduced alanine aminotransferase levels, insulin resistance, dyslipidemia, hepatic steatosis, and fibrosis markers in MASLD patients during phase II trials (4 mg dose) [252]. Pemafibrate (K‐877), a selective PPAR modulator, improved dyslipidemia, transaminase levels, and MASH pathology in animal studies [253], while also reducing hepatic inflammation and upregulating fatty acid oxidation genes in human trials [254, 255]. The broad spectrum PPAR agonist lanifibranor (IVA337) demonstrates greater efficacy over single/dual agonists in enhancing insulin sensitivity and reducing macrophage activation and fibrosis [256, 257]. Moreover, ACC inhibitors constitute another promising therapeutic strategy for MASLD by reducing hepatic steatosis and improving metabolic parameters [258]. The liver‐specific ACC1/2 dual inhibitor PF‐05221304 decreased DNL and steatosis in Western diet fed rats while improving MASH‐related inflammation and fibrosis [259].

Together, these approaches collectively target lipid flux dysregulation and secondarily dampening inflammation and fibrosis; moving forward, precision deployment—guided by mechanism—will be critical to clinical impact.

### 7.2 Targeting Cell Death

Programmed cell death constitutes a shared, targetable axis of liver injury across ALD and MASLD. In ALD, ethanol exposure robustly induces apoptosis and pyroptosis, whereas in MASLD, there are also an increasing number of treatment strategies for apoptosis, pyroptosis, ferroptosis, and cuproptosis. This section synthesizes therapeutic strategies.

Alcohol feeding induces apoptosis (evidenced by poly ADP‐ribose polymerase and CASP‐3 cleavage) and pyroptosis (evidenced by GSDMD cleavage) in the liver, during ALD pathogenesis. Research indicates that inhibition of CCL2 signaling using the dual CCR2/CCR5 inhibitor cenicriviroc (CVC) can prevent both apoptosis and pyroptosis [260]. Pyroptosis, a recently defined form of programmed cell death, is intricately linked to inflammatory cascades. Oligomeric proanthocyanidins (OPCs), polyphenols extracted from grape seeds, possess anti‐inflammatory and antioxidant properties. Studies reveal that OPCs are proposed to attenuate alcohol‐induced hepatocyte pyroptosis by scavenging ROS, thereby reducing MLKL expression and preventing the translocation of phosphorylated MLKL (P‐MLKL) to lysosomes where it forms pores. This inhibition consequently attenuates CTSB‐mediated NLRP3 inflammasome activation. These findings offer a potential therapeutic avenue utilizing natural plant constituents to reduce alcoholic liver injury [261]. Furthermore, Ampelopsis grossedentata extract has been found to confer protection against ALD by modulating the gut–liver axis and suppressing the TLR4/NF‐κB/MLKL‐mediated necroptosis pathway, while concurrently restoring intestinal barrier integrity [262].

Interventions targeting ferroptosis constitute an emerging therapeutic strategy for ALD. Studies demonstrate that the ferroptosis inhibitor ferrostatin‐1 effectively attenuates alcohol‐induced hepatic steatosis and lipid peroxidation, an effect linked to modulation of the FNDC3B–AMPK signaling axis and improved iron metabolism [58]. Notably, iron chelators reversed hepcidin downregulation and systemic iron dyshomeostasis resulting from ACSS2 deficiency, thereby suppressing hepatocellular ferroptosis and ameliorating alcohol‐induced liver injury [56]. Furthermore, supplementation with specific gut microbiota, such as *Lachnospiraceae bacterium*, or its metabolite N‐acetylglutamate (NAG), activates the NRF2 pathway and significantly inhibits ferroptosis, attenuating OxS and hepatic inflammation—highlighting the promise of probiotic‐based therapies [263]. These findings underscore the promise of targeting key nodes in ferroptosis—including lipid peroxidation, iron dysregulation, and antioxidant defenses—for the treatment of ALD.

Numerous therapeutic approaches have been developed to attenuate MASLD by targeting hepatocyte apoptosis and dysfunctional autophagy resulting from lipid metabolic dysregulation. For instance, administration of CASP inhibitors (such as GS‐9450, Emricasan, and VX‐166) to MASH mice has been shown to suppress hepatocyte apoptosis, thereby alleviating liver injury and fibrosis [264, 265, 266]. The blueberry‐derived monomer tectorigenin enhances hepatocyte autophagy, reduces the activation of the pyroptosis‐related protein GSDMD and the release of inflammatory mediators, ultimately alleviating MASH pathology [267]. Other autophagy‐targeting agents, including fluvastatin and glibenclamide, have also demonstrated therapeutic benefits in MASH [268, 269]. In terms of pyroptosis, inhibiting this process by targeting the NLRP3 inflammasome demonstrates translational potential. The triterpenoid compound Antcin A can inhibit the assembly and activation of NLRP3, thereby reducing the occurrence of pyroptosis [270]. Similar effects are seen with dieckol, MCC950 (NLRP3 inhibitor), and vitamin D, which alleviate TG/free fatty acids accumulation and improve liver injury while reducing MASLD scores [178, 271, 272]. Additionally, long noncoding RNA growth arrest‐specific transcription 5 (GAS5) inhibits NLRP3‐mediated pyroptosis by binding miR‐28a‐5p in MASLD hepatocytes [273].

Therapeutic strategies targeting ferroptosis have been validated as effective in MASLD. Studies using various interventions, such as ginkgolide B, thymosin β4, and quercetin, to inhibit ferroptosis have consistently shown attenuation of MASLD‐associated lipotoxicity, steatosis, and other hepatic injuries [274, 275, 276, 277]. In contrast, research on cuproptosis‐targeted therapy for MASLD is still scarce, likely due to its incompletely elucidated mechanisms. Current exploratory strategies are currently directed toward correcting copper metabolism disorders to prevent excessive copper accumulation, or on regulating key cuproptosis‐related enzymes (such as ferredoxin 1 and lipoic acid synthetase) and modulating the expression of cuproptosis‐associated genes to inhibit aberrant cell death, thereby mitigating MASLD‐related steatosis, inflammation, and liver damage [278, 279, 280].

Taken together, cell‐death‐directed interventions in ALD and MASLD converge on limiting hepatocellular loss while blunting downstream inflammatory and fibrotic responses. Signals of benefit with CCR2/CCR5 antagonism (CVC), polyphenolic antioxidants and gut–liver axis modulators in ALD, alongside CASP inhibitors (such as GS‐9450, Emricasan, and VX‐166), and NLRP3‐targeted approaches (MCC950, Antcin A, dieckol, vitamin D; lncRNA GAS5–miR‐28a‐5p axis) in MASLD, underscore translational relevance.

### 7.3 Targeting Cellular Senescence

Cellular senescence, increasingly recognized as a targetable driver of hepatic injury, contributes to the pathogenesis of ALD and MASLD. In these disorders, metabolic and OxS precipitate a SASP that amplifies sterile inflammation, immune dysregulation, and fibrogenic remodeling. Accordingly, therapeutic strategies that suppress the SASP, prevent acetaldehyde‐induced hepatocyte senescence, and enhance the clearance of senescent cells represent rational avenues to restore hepatic homeostasis; the subsequent section outlines the mechanistic rationale for targeting these pathways.

The pathological progression of ALD is closely associated with hepatocyte senescence. Targeting senescent pathways has consequently emerged as a promising therapeutic strategy. The natural flavonoid dihydromyricetin (DMY) ameliorates ALD by suppressing hepatocyte senescence. Mechanistically, DMY inhibits miR‐155‐5p production in KCs, leading to upregulation of hepatic SIRT1 expression. This upregulation mitigates mitochondrial voltage‐dependent anion channel 1 protein release and ROS accumulation, thereby blocking the SASP and ultimately inhibiting hepatocyte senescence [281]. Researchers demonstrated that nicotinamide, as an NAD⁺ precursor, can block acetaldehyde‐induced hepatocyte senescence within 24 h, significantly reducing hepatic lipid accumulation and enhancing hepatic functional parameters in mouse models. Its tolerability profile is superior to that of the traditional senolytic agent ABT‐263, although early intervention is crucial [282].

OxS represents a promising therapeutic target for regulating cellular aging processes. Results from various experimental models indicate that inhibiting OxS can effectively suppress cellular senescence [283, 284, 285]. NAD metabolism, AMPK, and SIRT proteins have been identified as potential molecular targets in the beneficial effects of OxS reduction on cellular senescence [286]. Specifically, restoring cellular NAD levels can prevent aging, and eliminating senescent cells or antagonizing SASP can improve NAD homeostasis [287]. In the context of MASLD, AMPK activation has been shown to mitigate hepatic OxS and steatosis [42, 288, 289]. Similarly, SIRT1 exerts protective effects by reducing lipid accumulation and OxS—particularly mitochondrial ROS production—thereby attenuating MASLD progression [290, 291].

In summary, current evidence identifies cellular senescence as a druggable nexus in both ALD and MASLD. Senomorphics that attenuate the SASP—for example, DMY acting via the KC miR‐155‐5p–SIRT1 axis—early restoration of NAD^+^ with nicotinamide to block acetaldehyde‐induced hepatocyte senescence, and activation of AMPK and SIRT1 pathways to mitigate hepatic OxS and steatosis collectively suppress senescence programs, with a more favorable tolerability profile than classical senolytics.

### 7.4 Targeting Gut Microbiota

Microbiota‐targeted therapies, including probiotics, prebiotics, postbiotics, fecal microbiota transplantation (FMT), and phage therapy, demonstrate substantial promise for alleviating ALD and MASLD.

Microbiome modulation exhibits considerable therapeutic potential in ALD. For instance, species of *Lachnospiraceae* elevate NAG levels through activation of the Kelch‐like ECH‐associated protein 1–NRF2 pathway, thereby inhibiting ferroptosis [263]. Supplementation with *Lactobacillus acidophilus* has been shown to restore alcohol‐induced disruptions in phenylalanine metabolism, enhance intestinal barrier function, and ameliorate alcoholic steatohepatitis [292]. Furthermore, composite probiotic formulations help preserve gut barrier integrity by upregulating mucus secretion and tight junction protein expression. This reduces hepatic LPS levels, suppresses TLR4/NF‐κB‐mediated inflammatory signaling, and modulates the AMPK–PPARα pathway to alleviate hepatic lipid accumulation [293]. In the realm of prebiotics, *Schisandra chinensis* polysaccharide (SCP) demonstrates promising potential for ALD intervention via modulation of the gut microbiota and tryptophan metabolism. SCP significantly enriches intestinal lactobacilli—particularly *Lactobacillus reuteri*—restores levels of indole derivatives capable of activating the aryl hydrocarbon receptor (AHR), enhances colonic AHR pathway activity, repairs intestinal barrier damage, reduces circulating LPS, and attenuates hepatic inflammation, OxS, and lipid accumulation [294]. Postbiotics also exhibit therapeutic value. For example, heat‐killed *Lactobacillus johnsonii* promotes liver repair by activating the nucleotide‐binding oligomerization domain‐containing protein 2–IL‐23–IL‐22 innate immune axis, while simultaneously enriching butyrate‐producing bacteria to maintain a low‐oxygen intestinal microenvironment [295].

FMT, aimed at restoring global gut microbial homeostasis, shows broad prospects in ALD management. Studies indicate that FMT improves hepatic functional parameters, prolongs survival, enhances the intestinal mucosal barrier, reduces bacterial translocation, alleviates hepatic inflammation, and delays fibrosis progression [296]. FMT also modulates metabolic pathways such as arachidonic acid and retinol metabolism, effectively ameliorating gut dysbiosis and metabolic disturbances in ALD models [297]. Notably, FMT derived from urolithin A (UA)‐treated mice effectively ameliorates ER stress via regulation of MUP1, achieving efficacy comparable to direct UA treatment [298]. Similarly, FMT from ellagic acid‐treated mice established a causal role for the reshaped microbiota and neuronal PAS domain protein 2 (NPAS2) in ALD improvement [299]. Nevertheless, the application of FMT remains largely experimental, warranting further clinical validation.

In terms of targeting pathogens, phage therapy specifically eliminates cytolysin‐producing *Enterococcus faecalis*, significantly reducing hepatic cytolysin levels [121]. Inhibiting the synthesis of polysialic acid capsules mediated by the *E. coli kpsM* gene can block immune evasion mechanisms and mitigate alcohol‐induced liver injury [122]. Targeting microbial metabolic pathways offers novel therapeutic approaches for ALD. Specifically, alcohol consumption disrupts phenylalanine metabolism by reducing *Lactobacillus acidophilus*, leading to accumulation of phenylalanine that inhibits intestinal alkaline phosphatase activity, promotes inflammation, and impairs gut barrier function. Intervention with *L. acidophilus* restores phenylalanine metabolism, strengthens intestinal integrity, and ameliorates alcohol‐induced steatohepatitis in mice. In addition, patients with AH exhibit elevated gut microbial trimethylamine (TMA) levels, while TMA lyase inhibitors mitigate liver injury by blocking TMA/TMAO production [300]. Engineered bacterial therapies also show promise: modified *Escherichia coli* Nissle 1917 and *Lactobacillus reuteri* enhance the production of tryptophan metabolites indole‐3‐acetic acid and lactate, respectively, activating type 3 innate lymphoid cells to secrete IL‐22. This upregulates the expression of intestinal Reg3 antimicrobial peptides and reduces bacterial translocation to the liver [301, 302]. Clinical studies further confirm that IL‐22 not only reduces inflammatory markers but is also significantly correlated with improved liver function scores [303].

It is well established that gut microbiota dysbiosis and its metabolites play a critical role in the pathogenesis of MASLD. Therefore, modulating the gut microbiota has emerged as a promising therapeutic strategy for MASLD. Probiotic formulations, typically combining *Lactobacillus* and *Bifidobacterium* strains, exert therapeutic effects through multiple mechanisms: (1) modulating gut microbiota composition; (2) influencing BAs metabolism; (3) preserving intestinal barrier integrity; (4) downregulating proinflammatory cytokines while promoting SCFA production; and (5) reducing OxS [304]. Probiotic interventions have been demonstrated to prevent MASLD [305]. *Bifidobacterium* breve CM02‐09T increased beneficial bacterium and SCFA producing bacteria (e.g., *Bacteroides, Faecalibacterium*) in mice. This intervention enhanced gut barrier integrity by upregulating tight junction proteins (occludin, zonula occludens‐1 [ZO‐1]), reduced systemic LPS and TLR4/NF‐κB activation, lowered hepatic inflammatory cytokines (TNF‐α, IL‐6), and improved lipid metabolism, ultimately attenuating MASLD progression [306]. Furthermore, inhibiting TLR adaptor molecule 1 (also known as TIR‐domain‐containing adapter‐inducing IFN‐β) in intestinal epithelial cells enriches the probiotic *Akkermansia muciniphila* to convert choline into betaine, thereby improving liver lipid metabolism and inflammation. Therefore, it significantly improved the MASH symptoms of mice [307].

Except for probiotics, treatment with Huanglian Wendan Decoction increased the abundance of *Akkermansia muciniphila*, raised the level of SCFAs in the gut, and activated the LKB1/AMPK pathway. This multipathway improvement in MASLD involves suppression of lipogenic genes (SREBP1c, FAS), upregulation of fatty acid oxidation genes (CPT1A, PPARα), reduction of inflammatory cytokines (IL‐6, IL‐1β, TNF‐α), and enhanced insulin sensitivity [308]. Similar studies employed the synergy of epigallocatechin gallate and taurine to regulate the composition of the gut microbiota by altering the abundance of *Bacteroidetes, Firmicutes*, and *Proteobacteria*, achieving the same therapeutic effect on MASLD [309]. Probiotics, traditional Chinese medicines, and bioactive compounds consistently attenuate MASLD by modulating gut microbiota, activating key signaling pathways, and correcting dysregulated lipid metabolism while mitigating hepatic inflammation.

In addition, at present, many studies have been conducted on prebiotics, synbiotics, postbiotics, engineered probiotics, or bacteriophages‐based therapies, and they are regarded as promising strategies for microbiota treatment [310, 311, 312, 313, 314]. Clinical trials have demonstrated FMT's potential to ameliorate MASLD by reshaping gut microbiota [315]. Notably, FMT carries potential risks, necessitating rigorous donor screening to minimize adverse effects. In addition, various new metabolic drugs represented by PPAR agonists, FXR agonists and thyroid hormone receptor β (THR‐β) agonists are also under development.

Microbiota‐targeted therapies offer a coherent path to disease modification in ALD and MASLD by restoring barrier function and microbial ecology, reshaping BA/SCFA pools, and dampening endotoxemia with TLR4/NF‐κB/NLRP3 signaling. Pathogen‐ and pathway‐specific strategies—phage depletion of cytolysin‐positive Enterococcus, blocking *E. coli* capsule‐mediated evasion, metabolic rewiring (phenylalanine; choline→betaine; TMA/TMAO), and IL‐22–LKB1/AMPK activation—converge to reduce steatosis, inflammation, and fibrogenic remodeling. Given FMT risks and context‐dependent β‐glucan effects, biomarker‐guided, standardized, combination regimens with host‐directed agents (PPAR, FXR, THR‐β) are warranted.

### 7.5 Immunomodulation

Immune dysregulation is not merely a by‐product but a causal amplifier of liver injury in both ALD and MASLD. Ethanol and lipotoxicity‐driven epithelial barrier disruption exposes the liver to gut‐derived PAMPs and sterile DAMPs, activating pattern‐recognition pathways and maladaptive cytokine/chemokine networks that drive progression from steatosis to steatohepatitis and fibrosis. Thus, targeting immunomodulation is justified to restore gut–liver homeostasis, modulate innate and adaptive responses (e.g., macrophage polarization, inflammasome signaling etc.), and promote tissue repair while preserving host defense—providing a complementary, mechanism‐based avenue beyond purely metabolic interventions.

Therapeutic strategies aimed at enhancing innate immune clearance of gut‐derived bacteria represent an emerging therapeutic avenue in ALD. CRIg, a receptor on liver macrophages that mediates phagocytosis of bacteria such as Enterococcus faecalis, is downregulated in ALD. This impairment contributes to reduced clearance of gut‐translocating pathobionts and exacerbates liver inflammation. Importantly, administration of soluble CRIg–Ig protein protected mice from ethanol‐induced steatohepatitis, highlighting its therapeutic potential [316].

This interaction leads to an upregulation of the inflammatory cytokine IL‐1β, promoting ethanol‐induced hepatocellular damage. Furthermore, studies suggest that blocking IL‐1β can prevent alcohol‐related steatohepatitis in mice, offering a potential therapeutic intervention for reducing ALD [317]. Composed of human IL‐22 fused to IgG2‐Fc, the recombinant protein F‐652 exerts its effects primarily through epithelial cell IL‐22R1 binding. It exhibits minimal impact on immune cells, protecting tissues from inflammation and injury and enhancing repair processes. Clinical development of F‐652 for ALD is promising; however, confirming its utility in sAH necessitates further trials [318]. While inflammation is central to ALD pathogenesis, its dual role in tissue damage and repair complicates therapeutic targeting. Future perspectives involve refining patient stratification based on inflammatory signatures and developing combination therapies that simultaneously intervene in multiple pathways. A deeper understanding of immune cell heterogeneity and interorgan communication is paramount for advancing precision medicine in ALD.

Targeting inflammatory pathways, corticosteroids remain the standard of care pharmacological intervention for severe AH, despite their limited long‐term efficacy. Studies reveal that ethanol decreases *Akkermansia* in mice, but IL‐22 treatment can elevate this symbiont, restoring alcohol‐reduced Reg3γ and α‐defensin levels, restoring intestinal barrier function, and reducing endotoxemia [319].

Current research on immunotherapy for MASLD can be categorized into two aspects: immune cells and immune signaling pathways. For instance, therapeutic agents targeting macrophage polarization have demonstrated efficacy [320]. A study revealed that MASLD involves upregulated macrophagic 17β‐hydroxysteroid dehydrogenase 7 (17β‐HSD7), driving proinflammatory M1 polarization. Fenretinide inhibits 17β‐HSD7 activity, suppressing M1 polarization, reducing TNF‐α and IL‐1β release, and enhancing lipid clearance in macrophages. Macrophage‐specific knockdown of 17β‐HSD7 replicated these protective effects, confirming its therapeutic potential [321].

STING is a key adaptor protein responsible for signal transduction, activation of transcription factors, and production of proinflammatory cytokines. It also serves as a critical link between innate and adaptive immunity [322]. Recent research has uncovered for the first time that RNF13 exerts a protective effect in MASH by promoting tripartite motif‐containing protein 29 (TRIM29) stability via K63‐linked ubiquitination, leading to STING degradation. This suppresses downstream NF‐κB/IRF3 activation and reduces hepatic proinflammatory cytokine and IFN production, thereby alleviating liver injury. The study supports targeting the RNF13–STING axis as a potential therapeutic strategy for MASH [323]. At present, immunotherapy for MASLD holds mechanistic promise for improving liver inflammation and lipid metabolism disorders.

Together, Immune dysregulation is a convergent, druggable driver in both ALD and MASLD. This section synthesizes immunomodulatory strategies that (i) recalibrate the gut–liver axis and innate sensing in ALD–corticosteroids for sAH; epithelial‐targeted IL‐22/F‐652 to restore barrier defenses (Akkermansia, Reg3γ, α‐defensins) and limit endotoxemia; CRIg‐based augmentation of bacterial clearance; and IL‐1β blockade for β‐glucan/CLEC7A‐driven inflammation and (ii) reprogram macrophage polarization in MASLD, including inhibition of macrophage 17β‐HSD7 with nilotinib and dampening STING signaling via the RNF13–TRIM29 axis. These approaches demonstrate that precise immunorecalibration can attenuate hepatic inflammation while preserving epithelial repair and microbial containment.

## 8 Ongoing Clinical Trials

The transition from basic research to clinical application in ALD and MASLD is rapidly evolving, with a growing number of therapeutic candidates entering various stages of clinical evaluation. Current strategies are strategically targeting core mechanisms such as disordered lipid metabolism, cellular senescence, gut–liver axis dysbiosis, and systemic inflammation. To provide a clear overview of this evolving landscape, the table below summarizes selected clinical‐stage investigations, organized by therapeutic approach, and details their respective clinical trial identifiers, phases, molecular targets, and primary objectives or findings (Table 5). These therapeutics, which target pivotal pathways, are poised to enter clinical practice and offer novel approaches for managing ALD and MASLD.

**TABLE 5:** Mechanistically targeted therapies in clinical development for ALD and MASLD.

Research phase	Therapeutic strategy/target	Core target/pathway	Main findings/objective summary
ALD			
Disordered lipid metabolism			
Phase 2 randomized controlled trial (NCT07046819)	Tirzepatide (GIP/GLP‐1 receptor agonist)	GIP receptor/GLP‐1 receptor	Evaluates the efficacy and safety of tirzepatide in reducing alcohol intake and improving liver health parameters over a 12‐week period in individuals with both alcohol use disorder and metabolic alcohol‐associated liver disease (MetALD).
Phase 2/3 randomized controlled trial (NCT04971577)	Simvastatin 40 mg (HMG‐CoA reductase inhibitor)	HMG‐CoA reductase/cholesterol synthesis	A randomized, double‐blind, placebo‐controlled trial to evaluate the efficacy of simvastatin in reducing liver fibrosis in patients with advanced fibrosis due to alcohol.
Not applicable (NCT06546384)	Semaglutide (GLP‐1 RA) and behavioral counseling	GLP‐1 receptor/mesolimbic dopamine system (reward pathway)	A randomized study assessing if semaglutide, combined with weight reduction counseling, leads to significantly higher alcohol abstinence rates compared with counseling alone in obese patients with fatty liver disease and alcohol use disorder. It also evaluates changes in liver parameters and uses the direct alcohol biomarker PEth for objective assessment.
Cellular senescence			
Phase 2a randomized controlled trial (NCT05639543)	INT‐787 (FXR agonist)	Farnesoid X receptor (FXR) signaling	A dose‐escalation, proof‐of‐concept study to evaluate the safety, tolerability, efficacy, and pharmacokinetics of the FXR agonist INT‐787 compared with placebo in subjects with severe alcohol‐associated hepatitis, to select optimal doses for future trials.
Gut dysbiosis			
Phase 3 randomized controlled trial (NCT04758806)	Fecal microbial transplantation (FMT) via upper GI tract	Gut microbiome–liver axis	Evaluates whether FMT improves survival in patients with severe alcoholic hepatitis (SAH) who are nonresponders to or ineligible for corticosteroid therapy, using microbiota from healthy donors.
Phase 2 randomized controlled trial (NCT05178069)	Lactobacillus Rhamnosus GG (Probiotic)	Gut–liver–brain axis/gut barrier and inflammation	A 6‐month trial testing the efficacy of the probiotic LGG versus placebo in treating alcohol use disorder (AUD) and alcoholic hepatitis. It aims to reduce heavy drinking, improve liver injury, and explore mechanistic markers of gut barrier function, inflammation, and gut‐brain communication.
Phase 1/2 randomized controlled trial (NCT05006430)	Fecal microbiota transplantation (PRIM‐DJ2727)	Gut microbiome–liver axis	A pilot study primarily evaluating the safety of oral, lyophilized FMT capsules in severe alcoholic hepatitis patients. Secondarily assesses trends in microbiome diversity and liver‐related outcomes after 4 weeks.
Immunomodulation			
Phase 2 randomized controlled trial (NCT06890039)	TAK‐242 (TLR4 inhibitor) and G‐CSF (filgrastim) combination	TLR4 signaling (inflammation) and hematopoietic stem cell mobilization	A double‐blind, randomized, placebo‐controlled study to evaluate the safety and efficacy of TAK‐242 (an immunomodulator) alone or in combination with G‐CSF (a stem cell mobilizer) in subjects with severe alcoholic hepatitis and acute‐on‐chronic liver failure. Primary endpoint is safety, with key secondary endpoints including changes in CLIF‐C OF score at Day 14.
Observational study (NCT02275195)	Analysis of immune cell frequency and function	Innate and adaptive immune system	Aims to characterize the relationship between immune cell dysfunction and disease progression in severe alcoholic hepatitis, and to assess the impact of standard treatments on these cells.

MASLD			
Disordered lipid metabolism			
Phase 2 randomized controlled trial (NCT04031729)	Low‐dose aspirin (81 mg)	Cyclooxygenase (COX)	Daily low‐dose aspirin (81 mg) for 6 months significantly reduced liver fat versus placebo, with some patients achieving a relative reduction in liver fat of ≥30%, all with a favorable safety profile.
Phase 2b randomized controlled trial (NCT04166773)	Tirzepatide (GIP/GLP‐1 receptor dual agonist)	GIP receptor/GLP‐1 receptor	After treatment with tirzepatide at weekly doses of 5, 10, and 15 mg, 51.8, 62.8, and 72.2% of patients achieved MASH resolution without worsening of fibrosis, respectively.
Phase 2a randomized controlled trial​(NCT04881760)	Retatrutide	GLP‐1R/GCGR/GIPR	Retatrutide (8/12 mg) for 24 weeks significantly reduced liver fat content (−81.7/−82.4% vs. −10.3% with placebo) and body weight versus placebo. A high proportion of patients (73/78%) achieved ≥30% absolute liver fat reduction.
Phase 2 randomized controlled trial (NCT05006885)	Pemvidutide (GLP‐1/glucagon dual receptor agonist)	GLP‐1 receptor/glucagon receptor	Pemvidutide significantly reduced liver fat content (−52.3 vs. −5.1%) and body weight (−9.2 vs. −0.5%) versus placebo, with a generally favorable safety profile.
Phase 2 randomized controlled trial (NCT04932512)	ION224 (DGAT2 antisense oligonucleotide inhibitor)	Diacylglycerol O‐acyltransferase 2 (DGAT2) and triglyceride synthesis pathway	ION224 demonstrated significant reductions in liver fat content, liver enzymes (ALT, AST), and improved histologic features of MASH compared with placebo, with an acceptable safety profile.
Not applicable (NCT06764056)	Combined semaglutide therapy and individualized nutritional approach	GLP‐1 receptor/dietary intervention	A clinical trial comparing the efficacy of three strategies over 1 year: individualized nutritional counseling alone, semaglutide medication alone, and their combination, for the treatment of obesity‐associated hepatic steatosis.
Observational study (NCT04720560)	Analysis of regulatory B cell function	Regulatory B cells/immune regulation	An observational study aiming to evaluate the role of regulatory B cells in the pathogenesis of metabolic‐associated fatty liver disease (MAFLD) by analyzing their frequency and phenotype in patient blood, and exploring their association with disease severity.
Not applicable (NCT06843148)	Low‐dose, postprandial, intermittent niacin (vitamin B3)	Niacin receptor (GPR109A)/adipose tissue fatty acid disposal	A mechanistic study using a randomized crossover design to investigate if niacin treatment reduces liver fat deposition and increases white adipose tissue storage of dietary fatty acids in MASLD patients, assessed via advanced metabolic imaging (PET/MRI).
Not applicable (NCT05558592)	Dietary supplementation with fresh oranges (polyphenols)	Polyphenol‐mediated metabolic regulation	A randomized study evaluating the effects of daily consumption of fresh “Tacle” oranges, rich in polyphenols (e.g., hesperidin), on improving carbohydrate and lipid metabolism in patients with MAFLD, compared with a balanced diet without oranges.
Not applicable (NCT07093346)	Low‐methoxy (LM) pectin supplementation	Gut microbiome–systemic inflammation axis	A randomized, placebo‐controlled dietary intervention investigating whether daily LM‐pectin for 6 weeks can reduce systemic inflammation and improve gut microbiota in MASLD patients, with impacts on metabolic indicators and liver health.
Gut dysbiosis			
Phase 4 randomized clinical trial (ChiCTR2200059043)	Silymarin (milk thistle extract)	Spirochaetota enrichment and modulation of associated bile acid and short‐chain fatty acid metabolism	Silymarin significantly reduced liver stiffness (measured by FibroScan) and serum LPS levels in MASLD patients, while increasing beneficial bacteria (Bifidobacterium, Lactobacillus) and decreasing pathogenic bacteria (Escherichia‐Shigella), correlating with improved liver function markers.
Not applicable (NCT07093346)	Low‐methoxy (LM) pectin supplementation	Gut microbiome–systemic inflammation axis	A randomized, placebo‐controlled dietary intervention investigating whether daily LM‐pectin for 6 weeks can reduce systemic inflammation and improve gut microbiota in MASLD patients, with impacts on metabolic indicators and liver health.
Immunomodulation			
Phase 2 randomized controlled trial (NCT05941910)	High‐dose coenzyme Q10 (CoQ10)	Mitochondrial electron transport chain and oxidative stress modulation	High‐dose CoQ10 (400 mg/day) significantly improved liver steatosis, endothelial and vascular function, and myocardial performance while reducing oxidative stress versus placebo.
Phase 2a randomized controlled trial (NCT03976401)	Efruxifermin (FGF21 analogue)	Fibroblast growth factor 21 (FGF21) signaling pathway	Weekly efruxifermin (28/50 mg) profoundly reduced liver fat (65–72%), with 47–68% of patients achieving ≥30% absolute reduction versus 0% on placebo, while also improving fibrosis markers, insulin sensitivity, and lipids.
Phase 2 randomized controlled trial (NCT05039450)	Efruxifermin (EFX) + GLP‐1 receptor ggonist	FGF21/GLP‐1 receptor signaling synergy	In patients on GLP‐1 RAs, adding efruxifermin (50/70 mg) for 12 weeks provided substantial additional reductions in liver fat (55.2–64.5%), with concurrent improvements in fibrosis markers, glycemic control, and lipids.
Not applicable (NCT06152250)	Characterization of intrahepatic inflammatory microenvironment	Intrahepatic immune cell phenotype and function	A pilot mechanistic study using liver fine‐needle aspiration and high‐definition technologies to define distinct inflammatory profiles in NAFL and NASH, aiming to identify new pathogenic drivers and therapeutic targets.

*Data source*: International Clinical Trials Registry Platform (via https://ichgcp.net).

## 9 Conclusion and Prospects

In this review, we systematically elucidate the shared and distinct molecular mechanisms underlying ALD and MASLD, particularly in lipid metabolism, cell death, cellular senescence, gut microbiota, and immune regulation, and summarize corresponding therapeutic intervention strategies. While these insights provide an important theoretical framework for precision‐based liver disease treatment, translating mechanistic insights into clinical practice remains a substantial challenge. These insights provide an important theoretical framework for precise liver disease treatment, and translating mechanism insights into clinical practice will bring new methods.

In the field of cell death, its critical role in driving the progression of both ALD and MASLD toward hepatitis and fibrosis has been clearly established. Targeting pyroptosis, ferroptosis, and cuproptosis has emerged as a highly promising new direction. For instance, inhibition of pyroptosis by targeting GSDMD or the NLRP3 inflammasome (e.g., with MCC950), as well as the use of ferroptosis inhibitors such as ferrostatin‐1, have shown beneficial effects in animal models. As a newly discovered form of cell death, the specific role and mechanisms of cuproptosis in liver diseases remain unclear. Actively exploring its interactions with mitochondrial metabolism and the immune microenvironment will be an important future focus. With a deeper understanding of cell death, future research should also explore other forms of cell death, such as NETosis (a form of inflammatory cell death in neutrophils) or immunogenic cell death, which is increasingly studied in liver cancer.

Regarding cellular senescence, current research primarily focuses on physiological aging; however, pathological senescence (induced by stressors such as alcohol and lipotoxicity) may exert a greater influence on disease progression in both ALD and MASLD. Senescent cells trigger inflammation and metabolic dysfunction through the SASP. Existing senotherapeutic strategies, including senolytics (which clear senescent cells, e.g., ABT263) and senomorphics (SASP inhibitors, e.g., DMY, acting via the miR‐155‐5p/SIRT1 axis), have shown efficacy in animal models. Nevertheless, their long‐term effects on tissue regeneration and immune function remain unclear. Due to the lack of universal markers for cellular senescence, future studies should aim to distinguish the characteristics and markers of physiological aging from those of disease‐specific senescence. It is also crucial to investigate the heterogeneity of senescence markers, as this would facilitate the precise elimination of pathogenic senescent cells without disrupting normal liver homeostasis. Developing drugs that target specific molecular pathways of senescence holds promise as a key focus for future ALD and MASLD treatment.

While probiotics and FMT have demonstrated modest benefits, their effects are often transient and subject to individual variation. Most currently developed probiotics are traditional strains of Lactobacillus or Bifidobacterium, with only a few candidate strains (e.g., Saccharomyces boulardii, Bacillus coagulans, Bacteroides ovatus, Lachnospiraceae bacterium, and Akkermansia muciniphila) showing efficacy in ALD and MASLD models. Future developments should progressively move toward precision interventions, such as engineered bacteria (Lactobacillus reuteri producing IL‐22, Escherichia coli Nissle 1917 producing tryptophan) or extracts of bioactive components (e.g., noni fruit phenolic‐rich extract, gallic acid, shikonin, hydroxytyrosol, obacunone, quercetin). These approaches can more precisely modulate host metabolism in ALD or MASLD by targeting microbial metabolites and key signaling pathways. Furthermore, the strategy of using phages to precisely eliminate cytolysin‐producing Enterococcus faecalis has been successfully validated, indicating the potential for large‐scale application of phages in the future to modulate specific bacterial populations in liver diseases. However, phage therapy also faces numerous uncertainties and challenges, including safety concerns, limited host range, immune clearance of specific phages, increased bacterial resistance, and difficulties in long‐term storage.

Similarly, immunomodulatory strategies must shift from broad‐spectrum anti‐inflammatory approaches to precise regulation of specific cells and signaling pathways. In research targeting specific immune cells, examples include modulating the balance of macrophage M1/M2 polarization (e.g., by inhibiting 17β‐HSD7), or eliminating pathological IL‐8‐producing neutrophil subsets. Targeting key immune molecules—such as inhibiting the cGAS–STING pathway, or targeting TLR4, NLRP3, or IL‐1β signaling—has shown promise for improved safety profiles in both ALD and MASLD. Furthermore, novel cellular therapies such as CAR‐T remain in their infancy for liver disease applications. The infusion of CAR‐T cells may carry risks of hepatotoxicity due to off‐target effects or cytokine release syndrome.

In summary, this review provides a fundamental theoretical basis for the shared molecular mechanisms of ALD and MASLD. Future efforts should focus on integrating additional animal models and conducting early clinical trials to translate these molecular insights into effective therapies, ultimately improving prognostic outcomes for both condition.

## Author Contributions

HKC was involved in the conception and design of the review and reviewed and revised the manuscript. YPT and YRH were responsible for writing the first draft and drawing the tables. YPT was responsible for drawing diagrams. YY revised the manuscript. All authors read and approved the final version of the manuscript.

## Funding

This study was funded by the National Key R&D Program of China (No. 2022YFA1305600 to H.C.) and the National Natural Science Foundation of China (No. 82470584 and No. 82000561 to H.C.)

## Conflicts of Interest

The authors declare no conflicts of interest.

## Ethics Statement

The authors have nothing to report.

## Data Availability

The authors have nothing to report.
